# Supplementary material for: BOSO: A novel feature selection algorithm for linear regression with high-dimensional data
Source: PLoS Comput Biol. 2022 May 31;18(5):e1010180. doi: 10.1371/journal.pcbi.1010180 (PMC9187084; doi:10.1371/journal.pcbi.1010180)
Supplement: S1 Appendix — (PDF) [file pcbi.1010180.s001.pdf]

S1 Appendix: Synthetic data generation and accuracy metrics

As detailed in Hastie et al. 2017 [1], synthetic data was obtained from a multivariate normal distribution in different settings, which depends on the number of instances (*n*), number of total available features (*p*), actual number of features contributing to the outcome (sparsity level, s) and their value (beta-type), autocorrelation level between different features (ρ) and signal-to-noise ratio (SNR level).

We describe below different **beta-type** considered, which the actual values of β_0:

- **Beta-type 1**: the s nonzero coefficients are equal to 1 and equally spaced in the p variables, while the rest are zero.
- **Beta-type 2**: the first s coefficients are nonzero and equal to 1, while the rest are zero.
- **Beta-type 3**: the first s coefficients are nonzero and equally distributed between 10 and 0.5, while the rest are zero.
- **Beta-type 5**: the first s coefficients are nonzero and equal to 1, while the rest are exponentially decaying with the following formula: $\beta_{0i} = 0.5^{i-s}; i = s+1, \ldots, p$.

Predictor autocorrelation level, ρ, was used to define the covariance matrix: $\Sigma_{ij} = \rho^{|i-j|}$. Three values were considered: 0, 0.35 and 0.7.

For **signal-to-noise ratio (SNR),** we selected 10 values equally distributed between 0.05 and 6.00 in logarithmic scale. The values are: 0.05, 0.09, 0.14, 0.25, 0.42, 0.71, 1.22, 2.07, 3.25 and 6.00. SNR was used to define the variance of the response variable, y, as detailed in Eq. (23) in the main text.

On the other hand, as defined in Hastie et al. 2017 [1], we used below the Relative test error, which is defined by:

$$\frac{\left(\hat{\beta} - \beta_0\right)^T \cdot \Sigma \cdot \left(\hat{\beta} - \beta_0\right) + \sigma^2}{\sigma^2}$$

, where $\hat{\beta}$ and β_0 represent the estimated and actual value of parameters for the different features considered, respectively; Σ is the covariance matrix of predictor features, whose entry (i,j) is equal $\rho^{(i-j)}$, being ρ the predictor correlation level; σ^2 is the variance of the response variable y. The variance σ^2 is related with the signal-to-noise ratio (SNR) as follows:

$$\sigma^2 = \frac{{\beta_0}^T \cdot \Sigma \cdot \beta_0}{SNR}$$

For this comparison below, once selected the features and optimal *K* with our MIQP and information criteria, we estimated the coefficients $\hat{\beta}$ only with training data, as done in Hastie et al. 2017 [1].

References

1. Hastie T, Tibshirani R, Tibshirani RJ. Extended comparisons of best subset selection, forward stepwise selection, and the lasso. *arXiv Prepr arXiv170708692. 2017.*
