# Supplementary figures and images for: BOSO: A novel feature selection algorithm for linear regression with high-dimensional data
Source: PLoS Comput Biol. 2022 May 31;18(5):e1010180. doi: 10.1371/journal.pcbi.1010180 (PMC9187084; doi:10.1371/journal.pcbi.1010180)

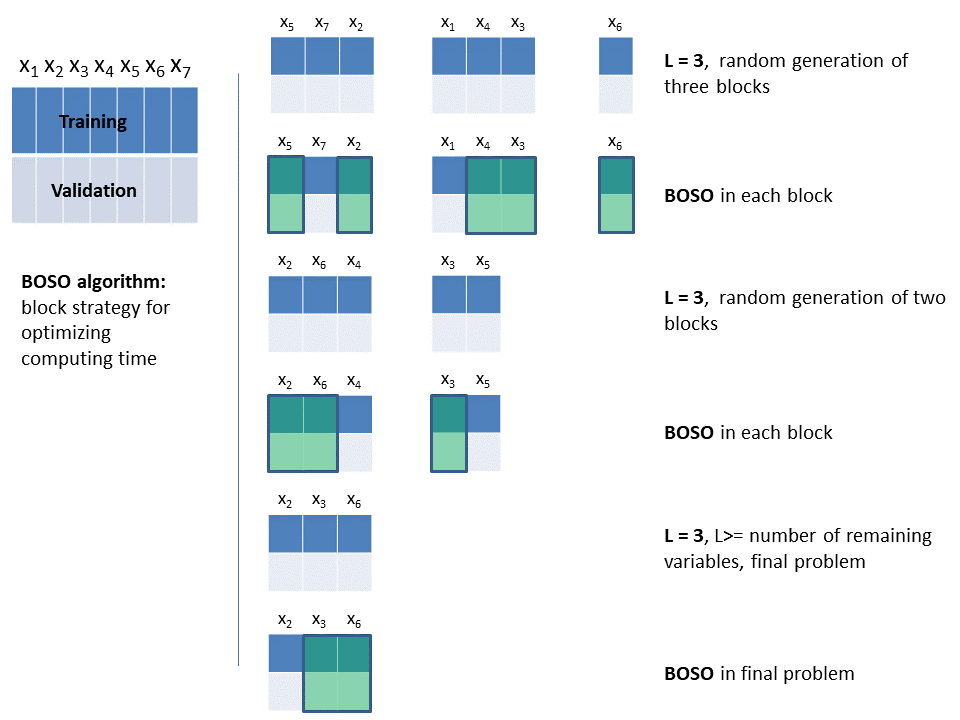

Supplement: S1 Fig — An example dataset with 7 features is split into training and validation sets. We defined random blocks of features of size L = 3. Green boxes represent the optimal selected features for a specific K value in certain block. In the first iteration, the dataset is separated in {X5, X7, X2}, {X1, X4, X3} and {X6}. Applying the BOSO algorithm to each block, we selected {X5, X2} in the first block, {X4, X3} in the secondo block and {X6} in the third block. Resulting variables are resampled again and randomly distributed into different blocks. In the second iteration, the blocks are {X2, X6, X4} and {X3, X5}. After BOSO, there are three remaining variables {X2, X6, X3}, which equals the block size. The final problem is re-solved, resulting in the optimal feature selection, which is {X3, X6} (TIF) [file pcbi.1010180.s014.tif]

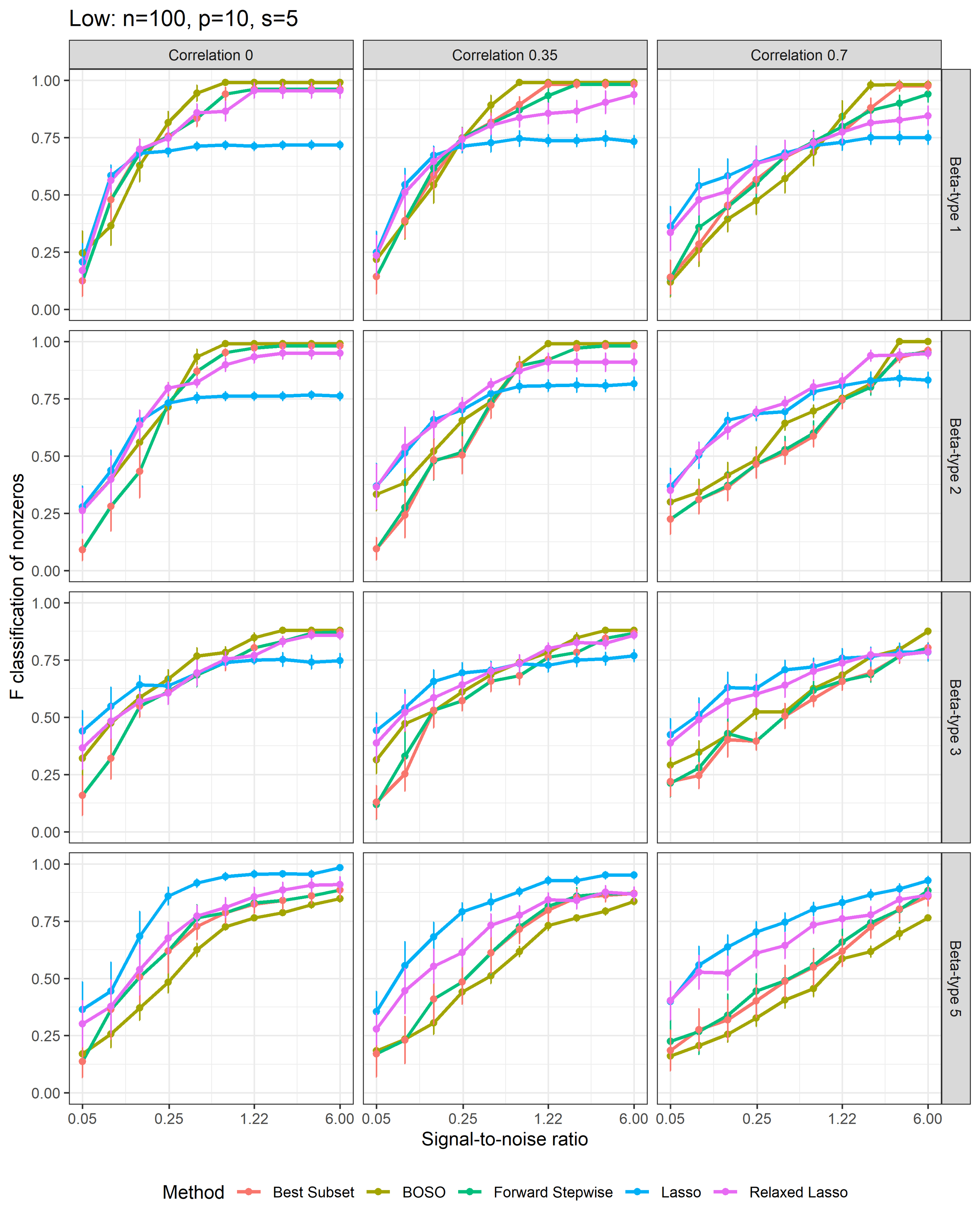

Supplement: S2 Fig — This accuracy metric is presented for the different feature selection methods (Best Subset, BOSO, Forward Stepwise, Lasso and Relaxed Lasso) and scenarios (according to Beta-type, autocorrelation levels and signal-to-noise ratio (SNR) levels) considered in the main text. S1 Appendix provides full details of the different situations considered. Points and error bars represent the mean and standard deviation in 10 random simulations, respectively. Note here that n is the number of instances, p is the total available features and s is the actual number of features contributing to the response variable. (TIF) [file pcbi.1010180.s015.tif]

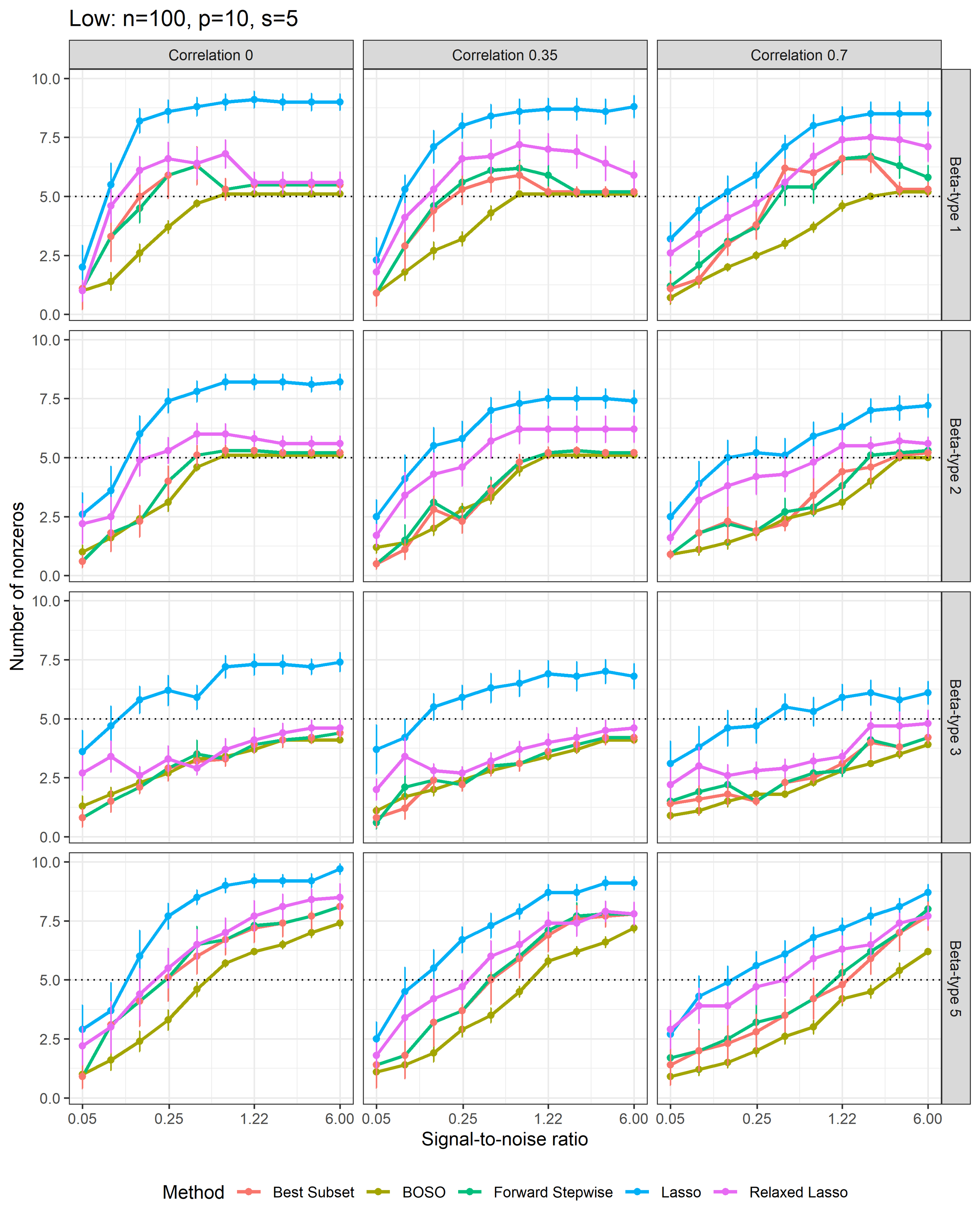

Supplement: S3 Fig — This accuracy metric is presented for the different feature selection methods (Best Subset, BOSO, Forward Stepwise, Lasso and Relaxed Lasso) and scenarios (according to Beta-type, autocorrelation levels and signal-to-noise ratio (SNR) levels) considered in the main text. S1 Appendix provides full details of the different situations considered. Points and error bars represent the mean and standard deviation in 10 random simulations, respectively. Note here that n is the number of instances, p is the total available features and s is the actual number of features contributing to the response variable. Dotted line represents the actual number of features. (TIF) [file pcbi.1010180.s016.tif]

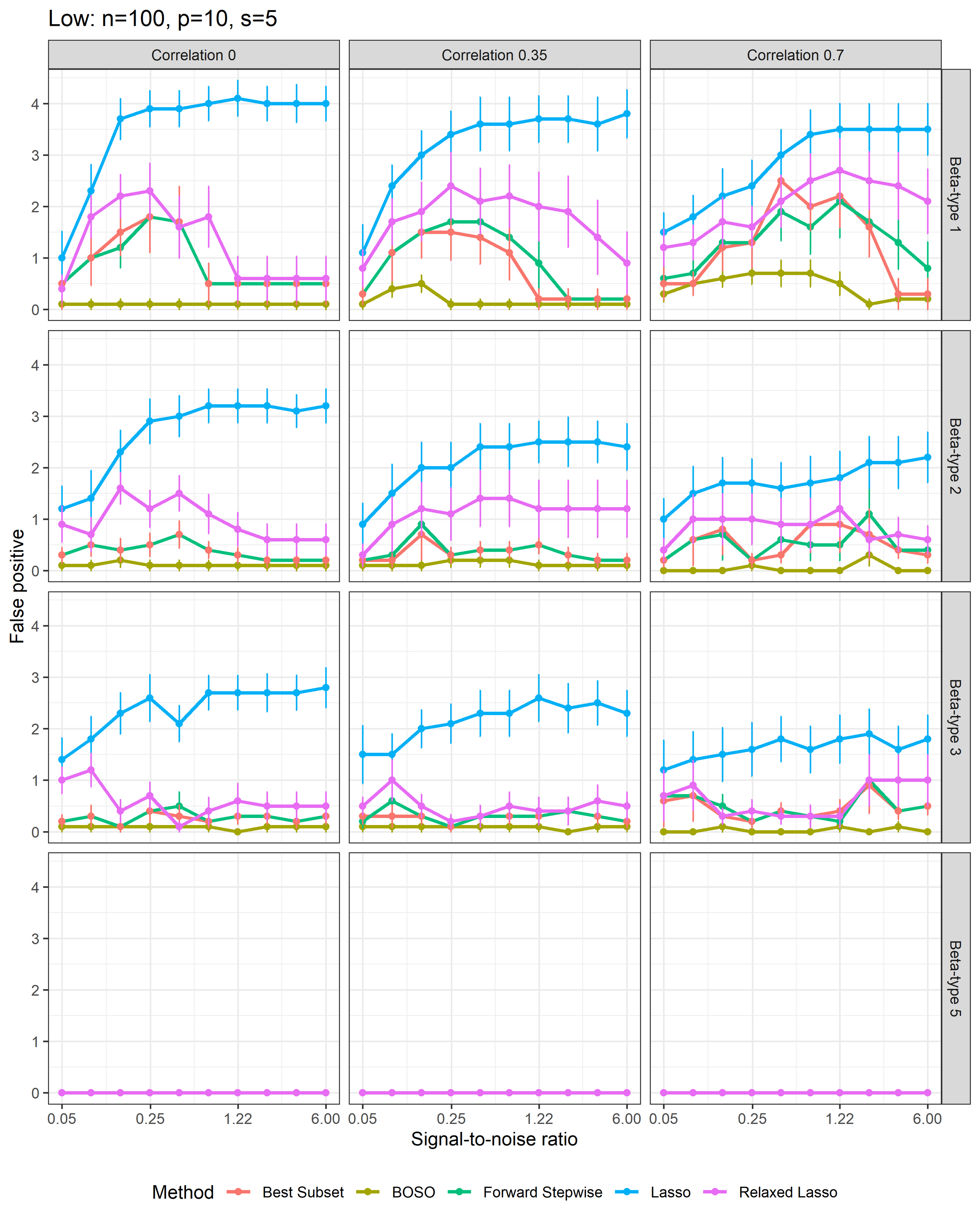

Supplement: S4 Fig — This accuracy metric is presented for the different feature selection methods (Best Subset, BOSO, Forward Stepwise, Lasso and Relaxed Lasso) and scenarios (according to Beta-type, autocorrelation levels and signal-to-noise ratio (SNR) levels) considered in the main text. S1 Appendix provides full details of the different situations considered. Points and error bars represent the mean and standard deviation in 10 random simulations, respectively. Note here that n is the number of instances, p is the total available features and s is the actual number of features contributing to the response variable. (TIF) [file pcbi.1010180.s017.tif]

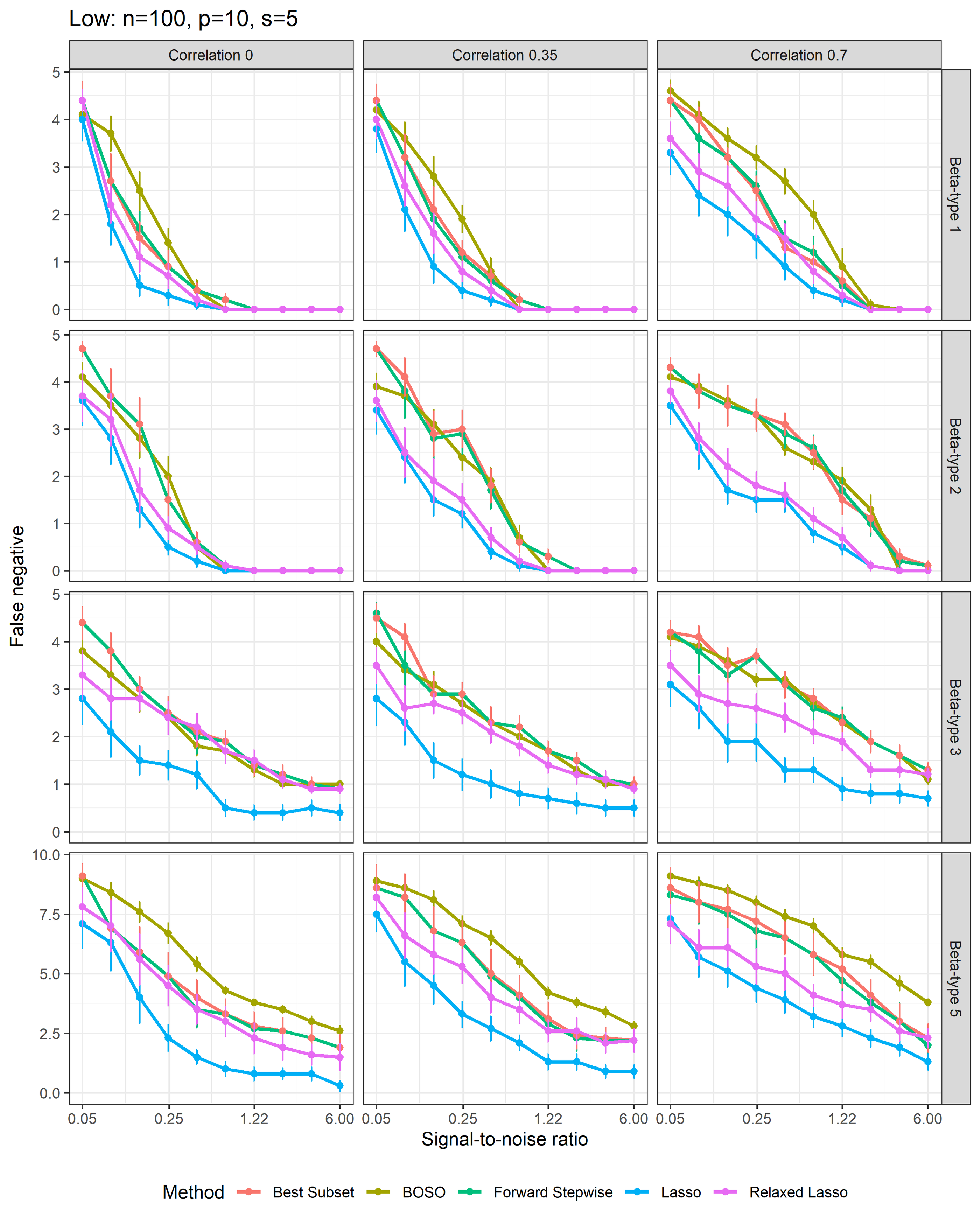

Supplement: S5 Fig — This accuracy metric is presented for the different feature selection methods (Best Subset, BOSO, Forward Stepwise, Lasso and Relaxed Lasso) and scenarios (according to Beta-type, autocorrelation levels and signal-to-noise ratio (SNR) levels) considered in the main text. S1 Appendix provides full details of the different situations considered. Points and error bars represent the mean and standard deviation in 10 random simulations, respectively. Note here that n is the number of instances, p is the total available features and s is the actual number of features contributing to the response variable. (TIF) [file pcbi.1010180.s018.tif]

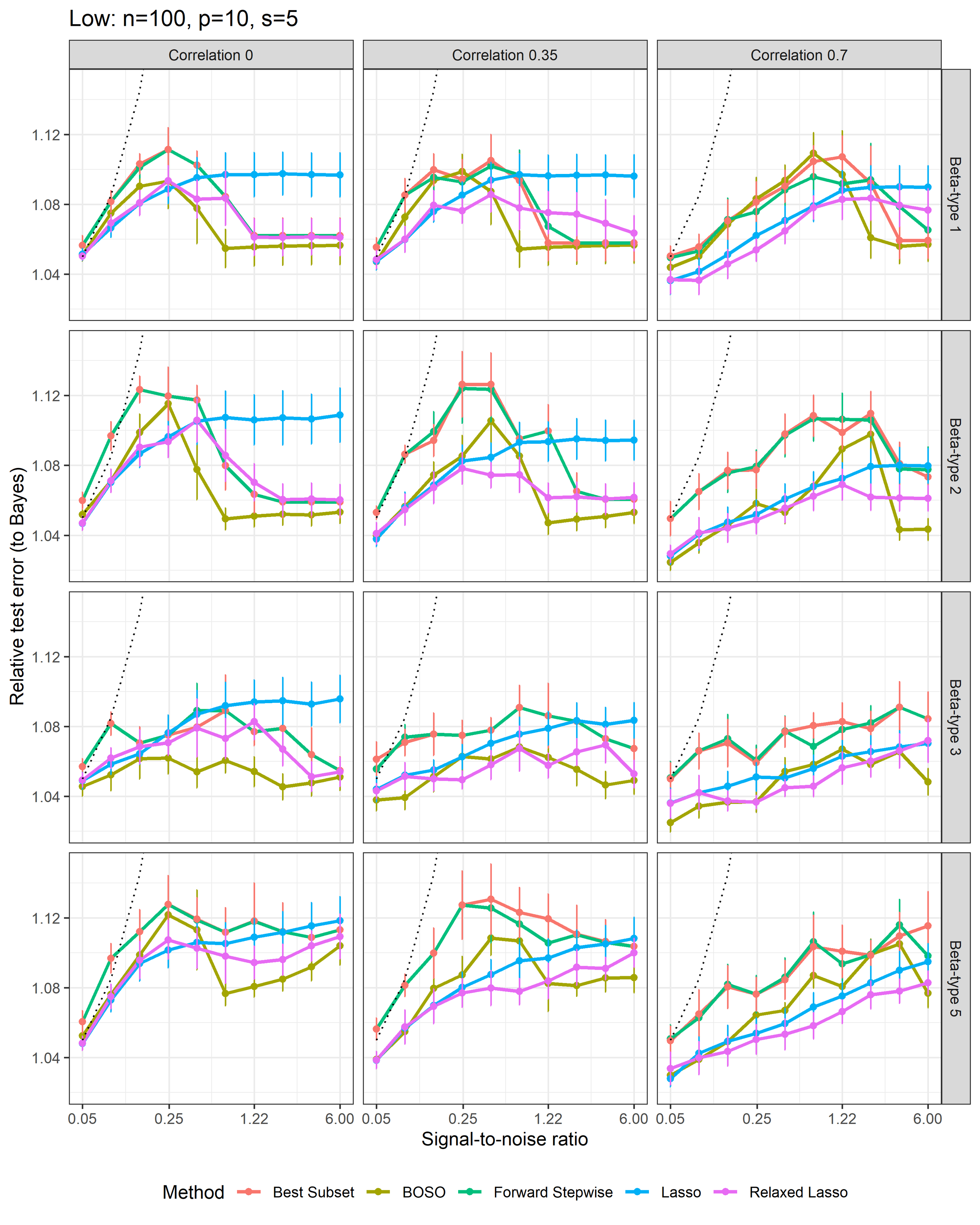

Supplement: S6 Fig — This accuracy metric is presented for the different feature selection methods (Best Subset, BOSO, Forward Stepwise, Lasso and Relaxed Lasso) and scenarios (according to Beta-type, autocorrelation levels and signal-to-noise ratio (SNR) levels) considered in the main text. S1 Appendix provides full details of the different situations considered. Points and error bars represent the mean and standard deviation in 10 random simulations, respectively. Note here that n is the number of instances, p is the total available features and s is the actual number of features contributing to the response variable. Dotted curve represents the results for the null model. (TIF) [file pcbi.1010180.s019.tif]

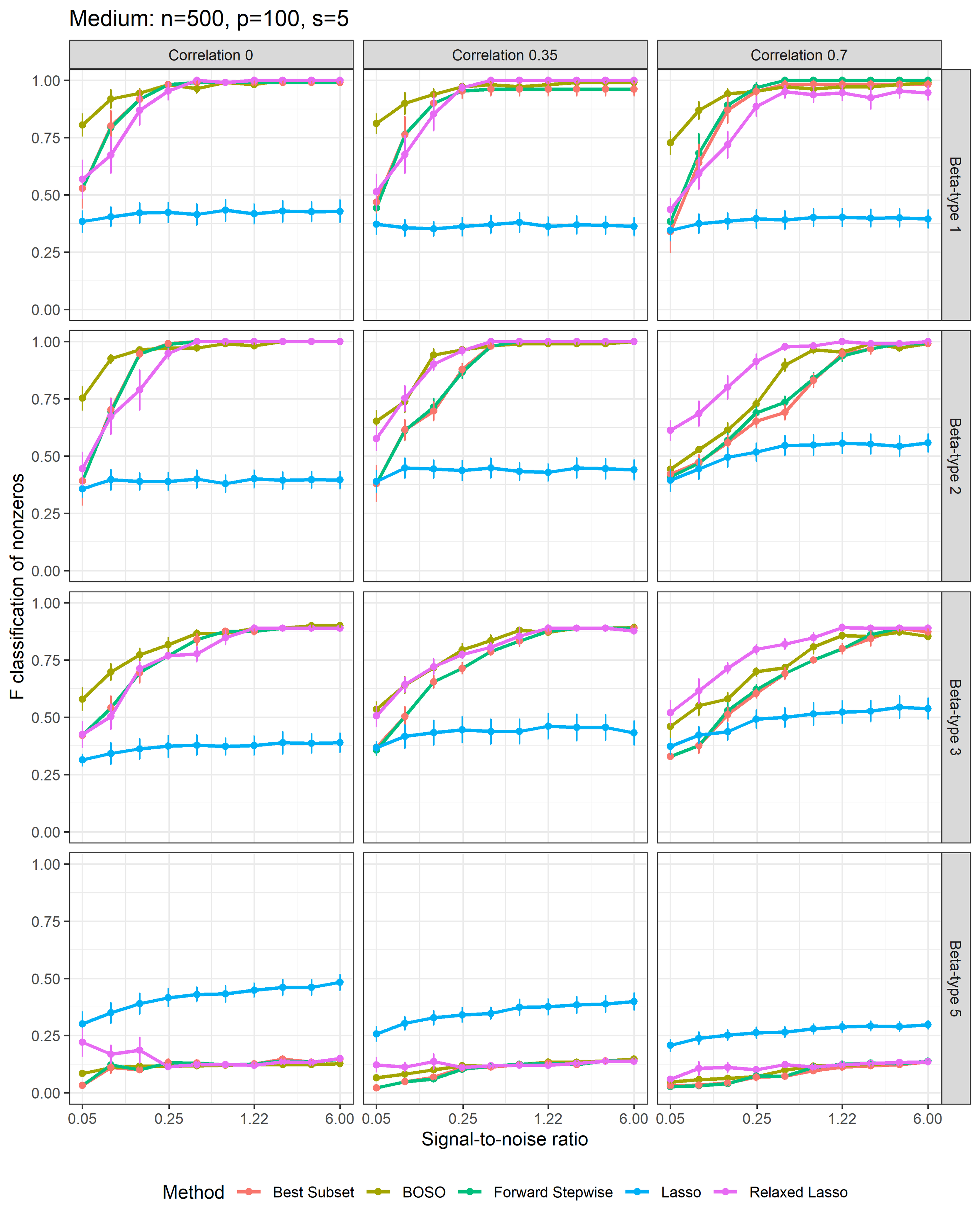

Supplement: S7 Fig — This accuracy metric is presented for the different feature selection methods (Best Subset, BOSO, Forward Stepwise, Lasso and Relaxed Lasso) and scenarios (according to Beta-type, autocorrelation levels and signal-to-noise ratio (SNR) levels) considered in the main text. S1 Appendix provides full details of the different situations considered. Points and error bars represent the mean and standard deviation in 10 random simulations, respectively. Note here that n is the number of instances, p is the total available features and s is the actual number of features contributing to the response variable. (TIF) [file pcbi.1010180.s020.tif]

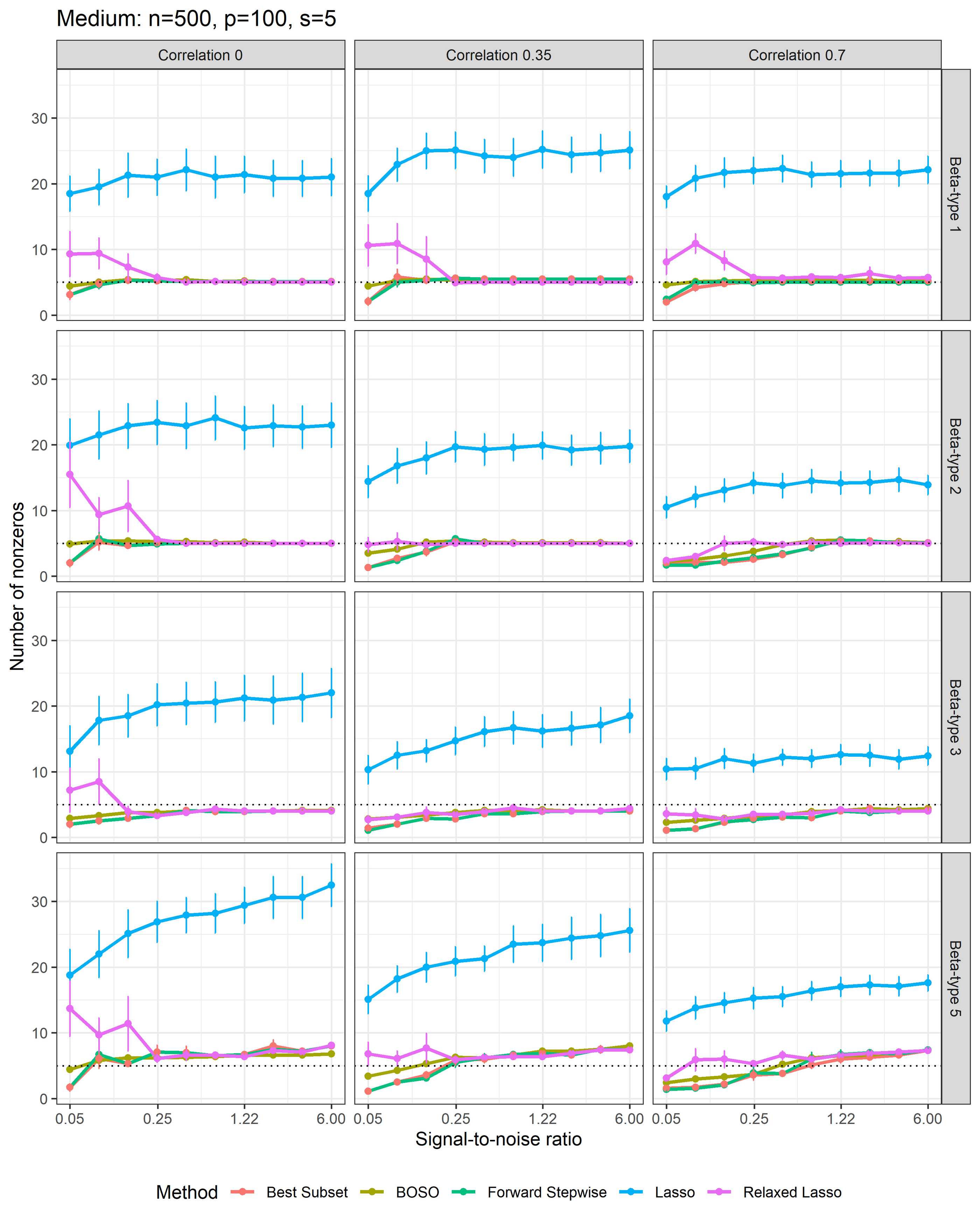

Supplement: S8 Fig — This accuracy metric is presented for the different feature selection methods (Best Subset, BOSO, Forward Stepwise, Lasso and Relaxed Lasso) and scenarios (according to Beta-type, autocorrelation levels and signal-to-noise ratio (SNR) levels) considered in the main text. S1 Appendix provides full details of the different situations considered. Points and error bars represent the mean and standard deviation in 10 random simulations, respectively. Note here that n is the number of instances, p is the total available features and s is the actual number of features contributing to the response variable. Dotted line represents the actual number of features. (TIF) [file pcbi.1010180.s021.tif]

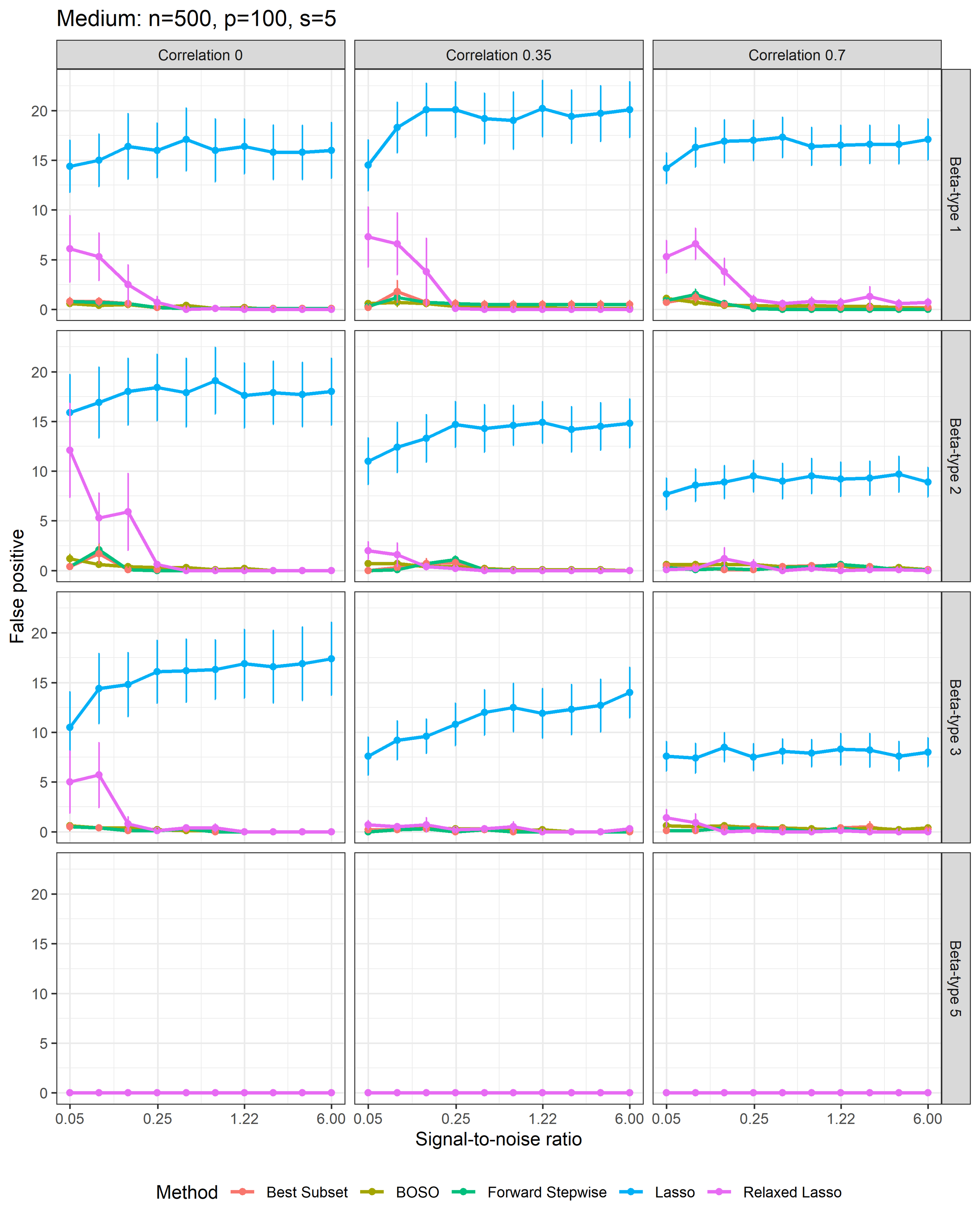

Supplement: S9 Fig — This accuracy metric is presented for the different feature selection methods (Best Subset, BOSO, Forward Stepwise, Lasso and Relaxed Lasso) and scenarios (according to Beta-type, autocorrelation levels and signal-to-noise ratio (SNR) levels) considered in the main text. S1 Appendix provides full details of the different situations considered. Points and error bars represent the mean and standard deviation in 10 random simulations, respectively. Note here that n is the number of instances, p is the total available features and s is the actual number of features contributing to the response variable. (TIF) [file pcbi.1010180.s022.tif]

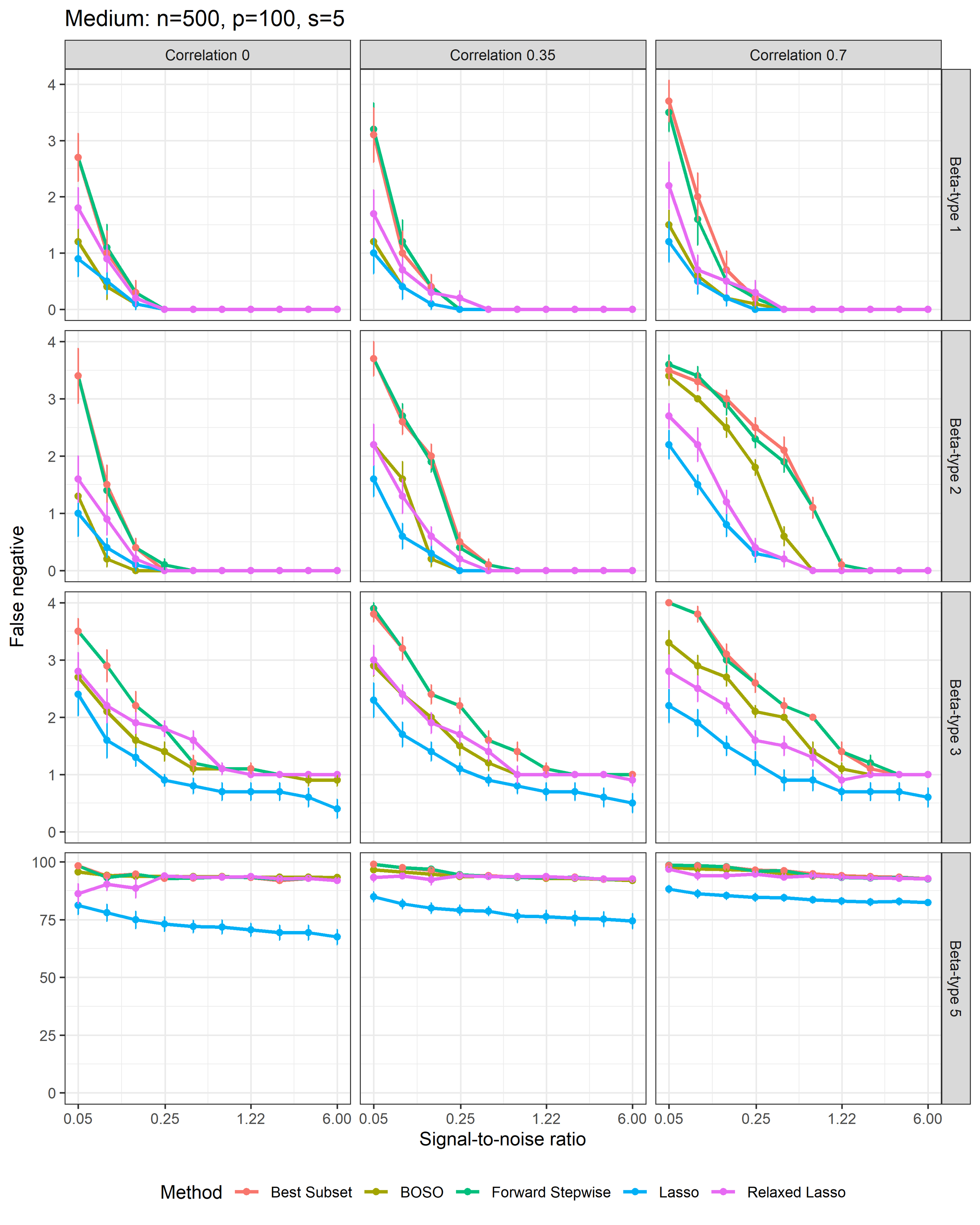

Supplement: S10 Fig — This accuracy metric is presented for the different feature selection methods (Best Subset, BOSO, Forward Stepwise, Lasso and Relaxed Lasso) and scenarios (according to Beta-type, autocorrelation levels and signal-to-noise ratio (SNR) levels) considered in the main text. S1 Appendix provides full details of the different situations considered. Points and error bars represent the mean and standard deviation in 10 random simulations, respectively. Note here that n is the number of instances, p is the total available features and s is the actual number of features contributing to the response variable. (TIF) [file pcbi.1010180.s023.tif]

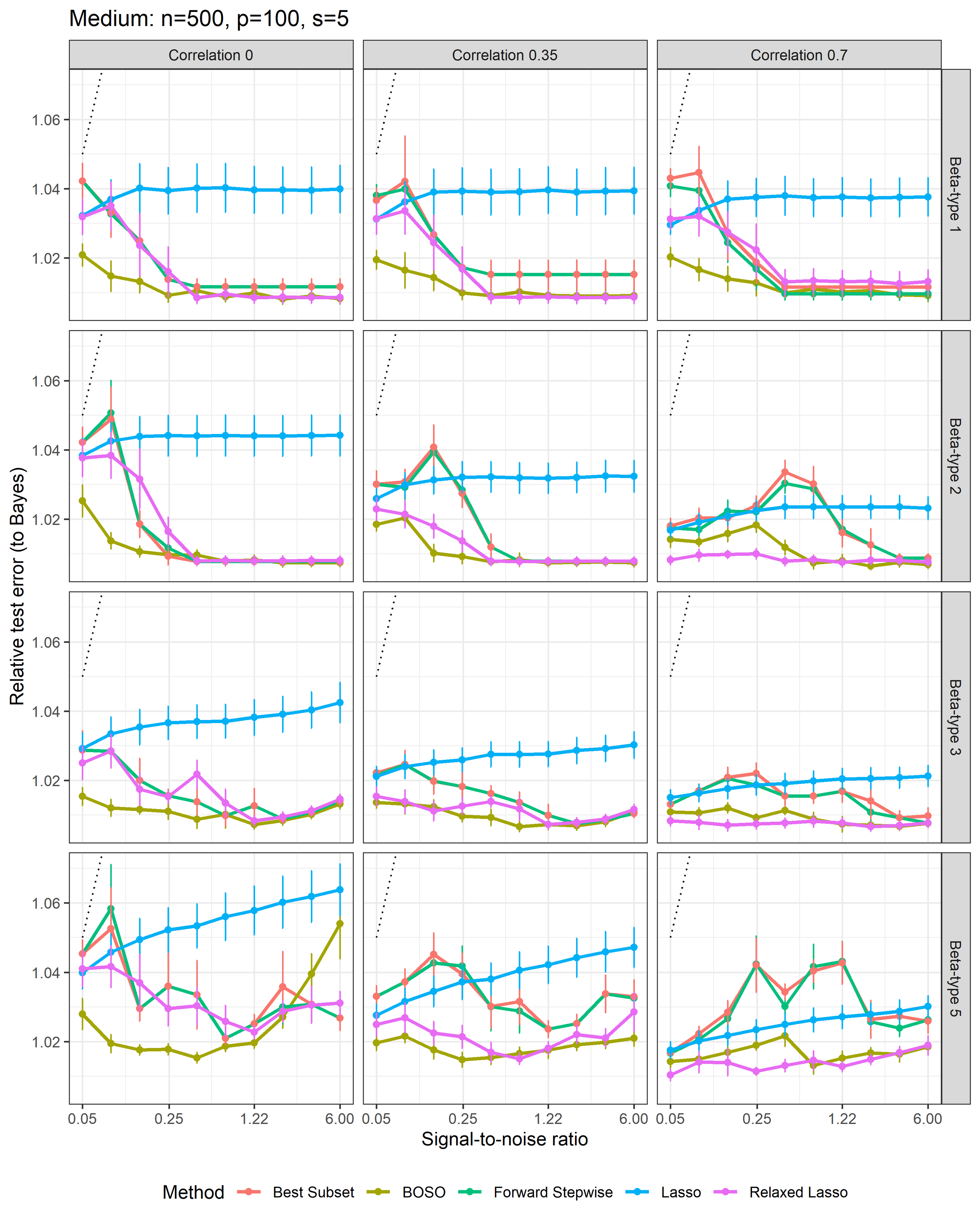

Supplement: S11 Fig — This accuracy metric is presented for the different feature selection methods (Best Subset, BOSO, Forward Stepwise, Lasso and Relaxed Lasso) and scenarios (according to Beta-type, autocorrelation levels and signal-to-noise ratio (SNR) levels) considered in the main text. S1 Appendix provides full details of the different situations considered. Points and error bars represent the mean and standard deviation in 10 random simulations, respectively. Note here that n is the number of instances, p is the total available features and s is the actual number of features contributing to the response variable. Dotted line represents the results for the null model. (TIF) [file pcbi.1010180.s024.tif]

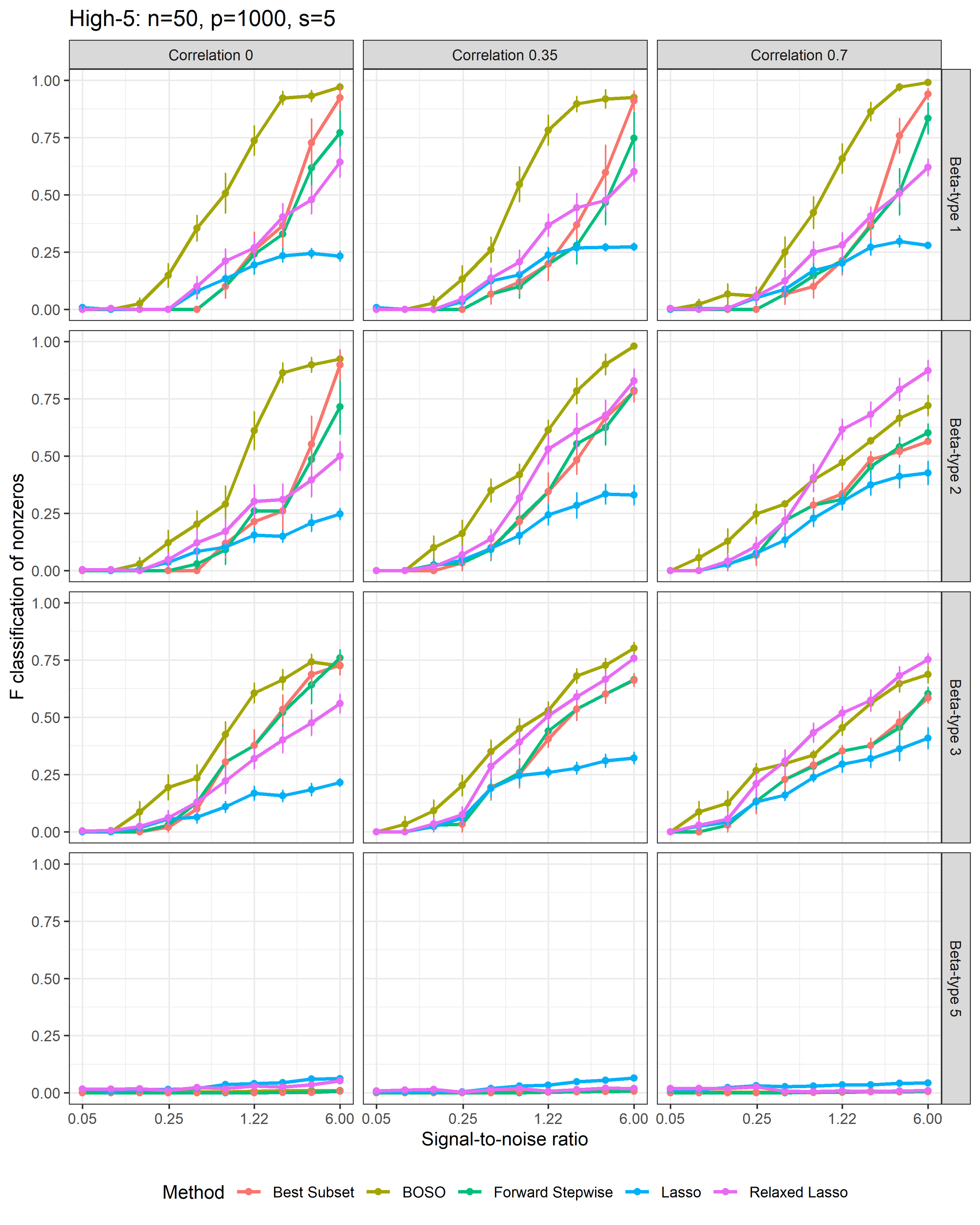

Supplement: S12 Fig — This accuracy metric is presented for the different feature selection methods (Best Subset, BOSO, Forward Stepwise, Lasso and Relaxed Lasso) and scenarios (according to Beta-type, autocorrelation levels and signal-to-noise ratio (SNR) levels) considered in the main text. S1 Appendix provides full details of the different situations considered. Points and error bars represent the mean and standard deviation in 10 random simulations, respectively. Note here that n is the number of instances, p is the total available features and s is the actual number of features contributing to the response variable. (TIF) [file pcbi.1010180.s025.tif]

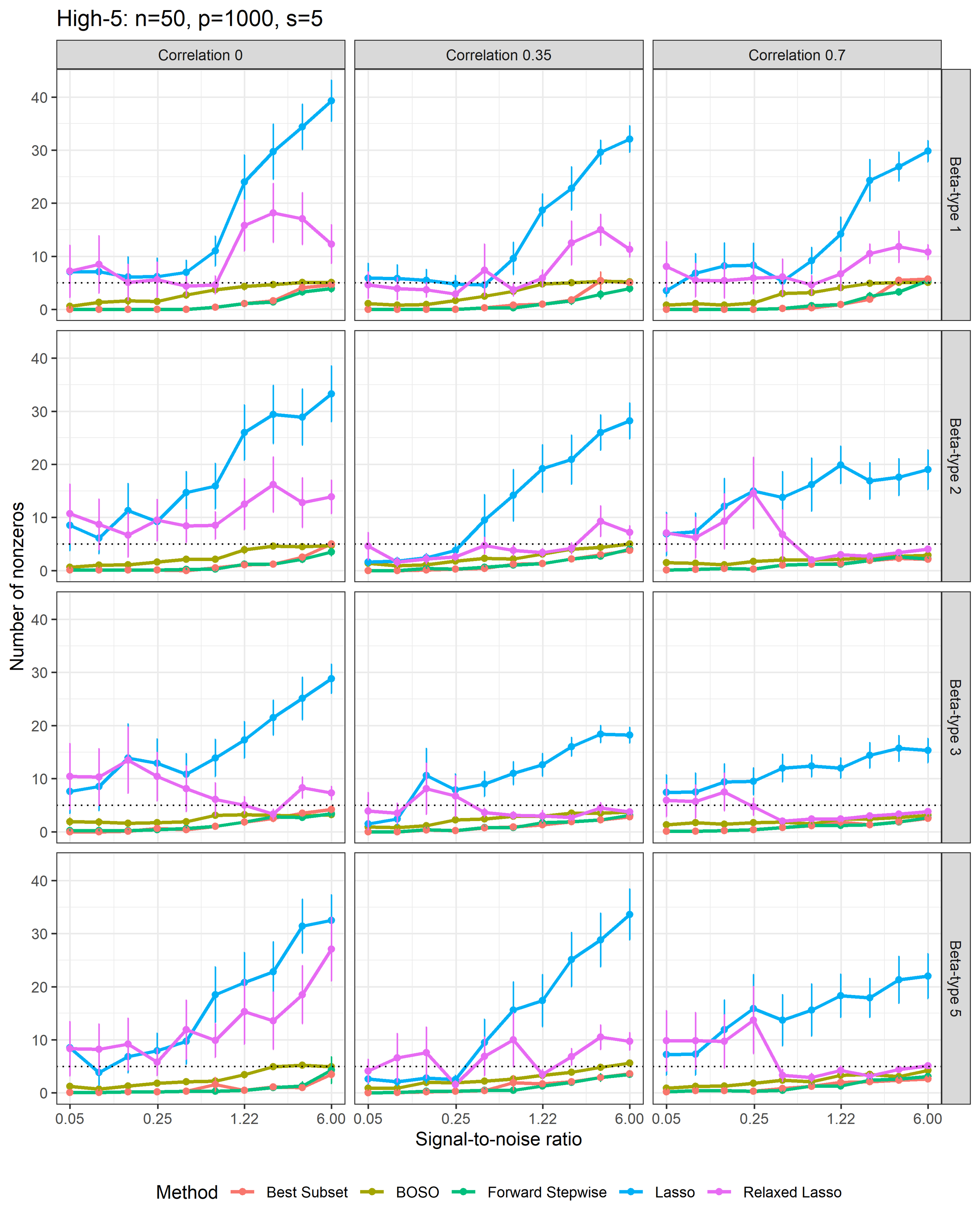

Supplement: S13 Fig — This accuracy metric is presented for the different feature selection methods (Best Subset, BOSO, Forward Stepwise, Lasso and Relaxed Lasso) and scenarios (according to Beta-type, autocorrelation levels and signal-to-noise ratio (SNR) levels) considered in the main text. S1 Appendix provides full details of the different situations considered. Points and error bars represent the mean and standard deviation in 10 random simulations, respectively. Note here that n is the number of instances, p is the total available features and s is the actual number of features contributing to the response variable. Dotted line represents the actual number of features. (TIF) [file pcbi.1010180.s026.tif]

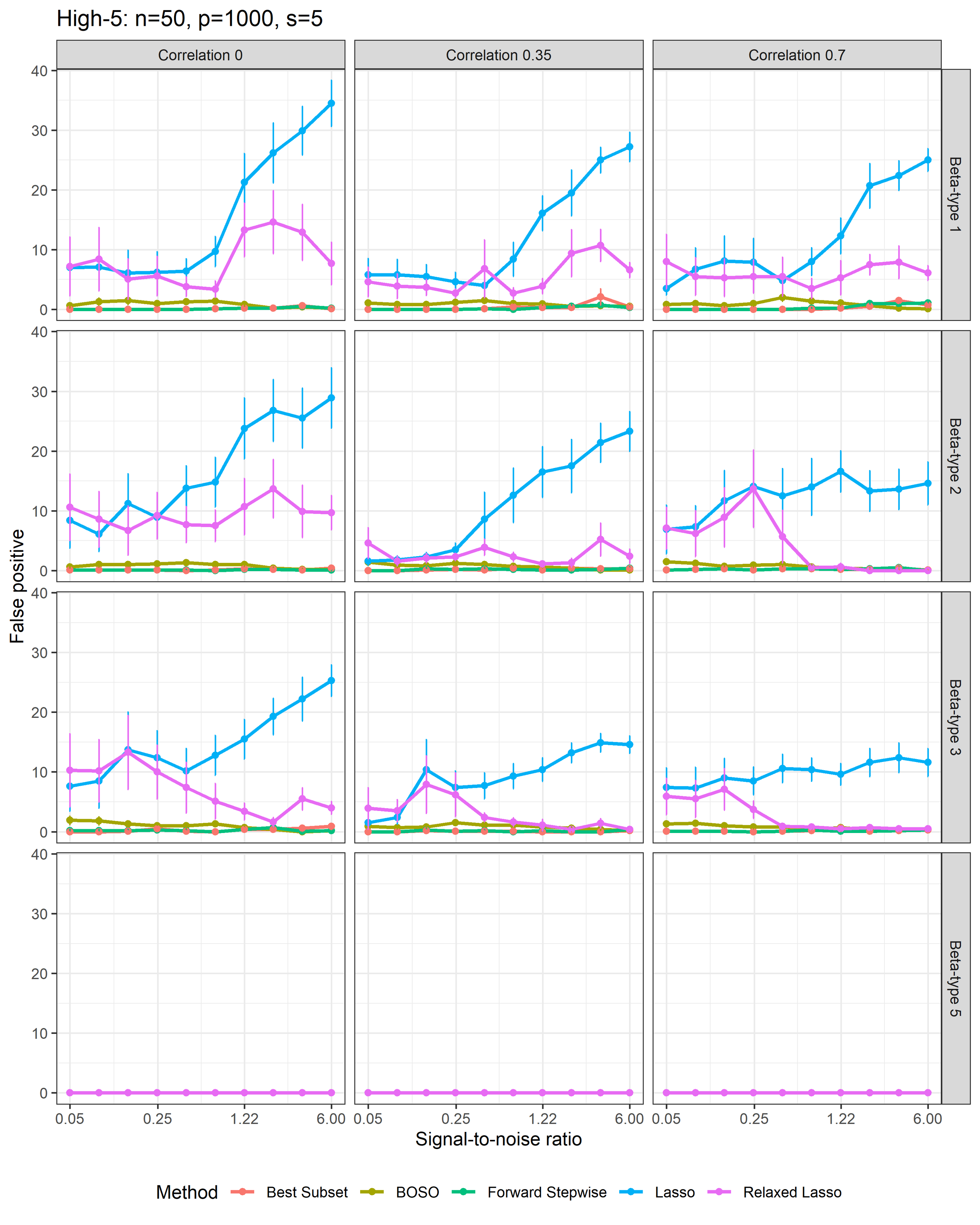

Supplement: S14 Fig — This accuracy metric is presented for the different feature selection methods (Best Subset, BOSO, Forward Stepwise, Lasso and Relaxed Lasso) and scenarios (according to Beta-type, autocorrelation levels and signal-to-noise ratio (SNR) levels) considered in the main text. S1 Appendix provides full details of the different situations considered. Points and error bars represent the mean and standard deviation in 10 random simulations, respectively. Note here that n is the number of instances, p is the total available features and s is the actual number of features contributing to the response variable. (TIF) [file pcbi.1010180.s027.tif]

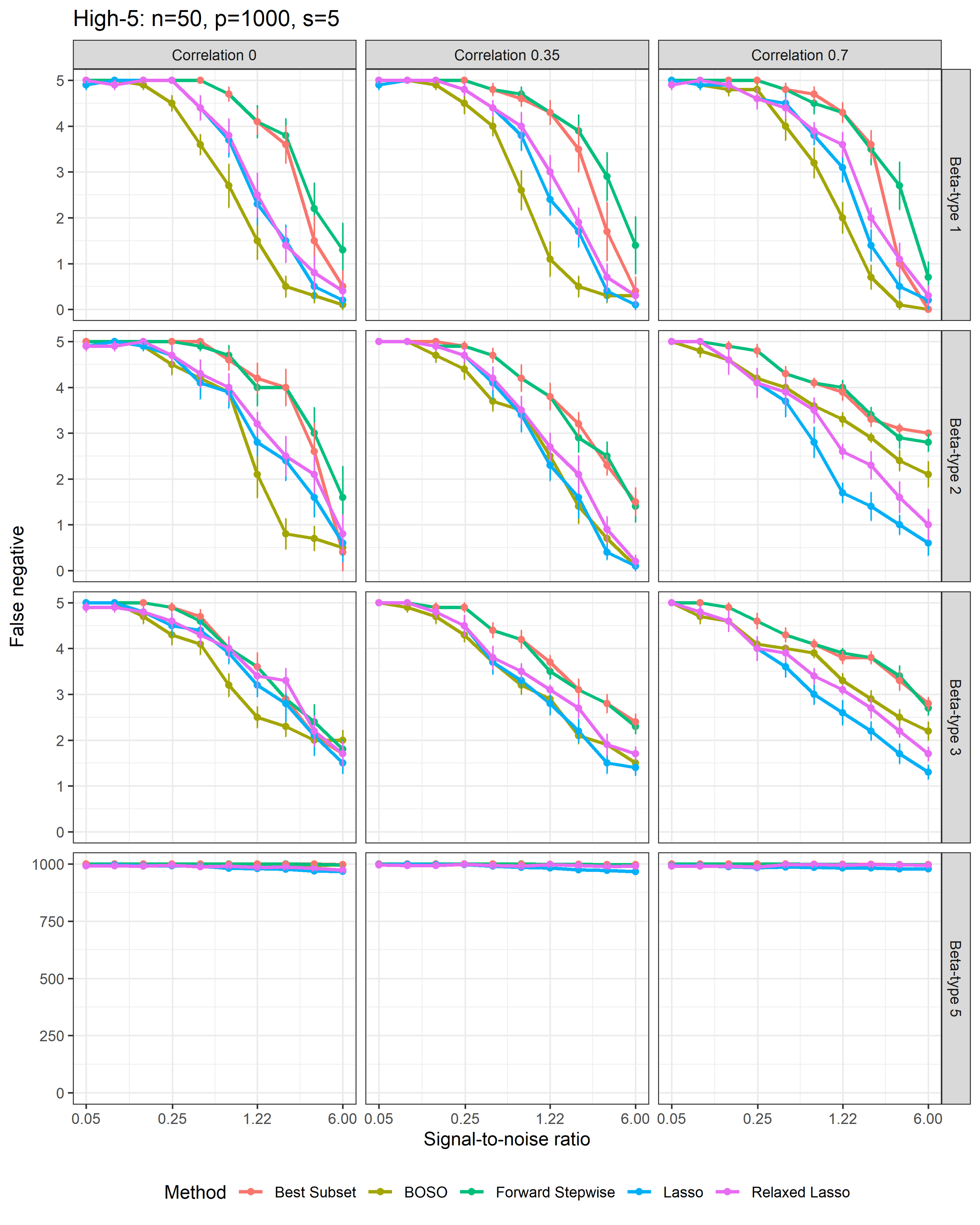

Supplement: S15 Fig — This accuracy metric is presented for the different feature selection methods (Best Subset, BOSO, Forward Stepwise, Lasso and Relaxed Lasso) and scenarios (according to Beta-type, autocorrelation levels and signal-to-noise ratio (SNR) levels) considered in the main text. S1 Appendix provides full details of the different situations considered. Points and error bars represent the mean and standard deviation in 10 random simulations, respectively. Note here that n is the number of instances, p is the total available features and s is the actual number of features contributing to the response variable. (TIF) [file pcbi.1010180.s028.tif]

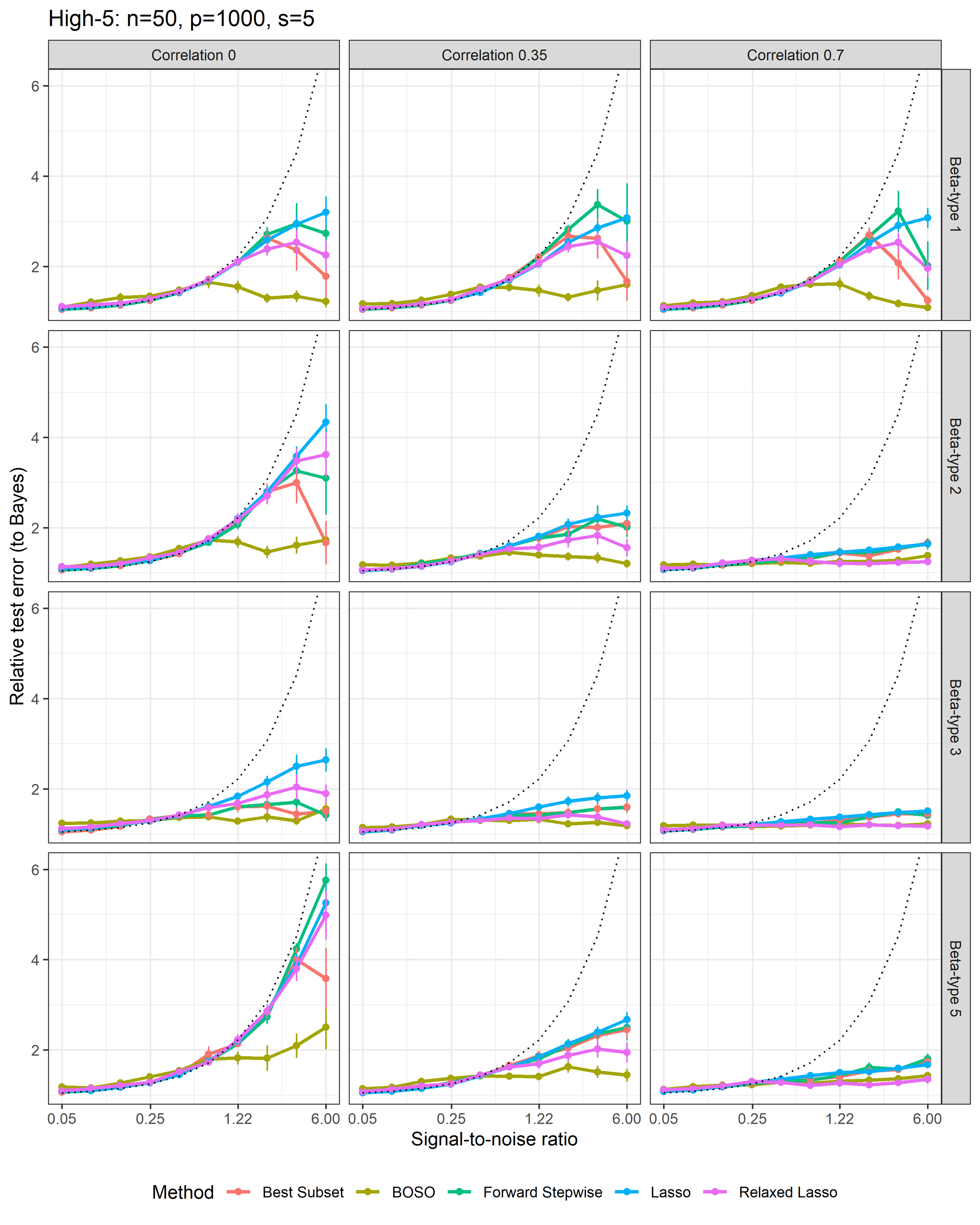

Supplement: S16 Fig — This accuracy metric is presented for the different feature selection methods (Best Subset, BOSO, Forward Stepwise, Lasso and Relaxed Lasso) and scenarios (according to Beta-type, autocorrelation levels and signal-to-noise ratio (SNR) levels) considered in the main text. S1 Appendix provides full details of the different situations considered. Points and error bars represent the mean and standard deviation in 10 random simulations, respectively. Note here that n is the number of instances, p is the total available features and s is the actual number of features contributing to the response variable. Dotted curve represents the results for the null model. (TIF) [file pcbi.1010180.s029.tif]

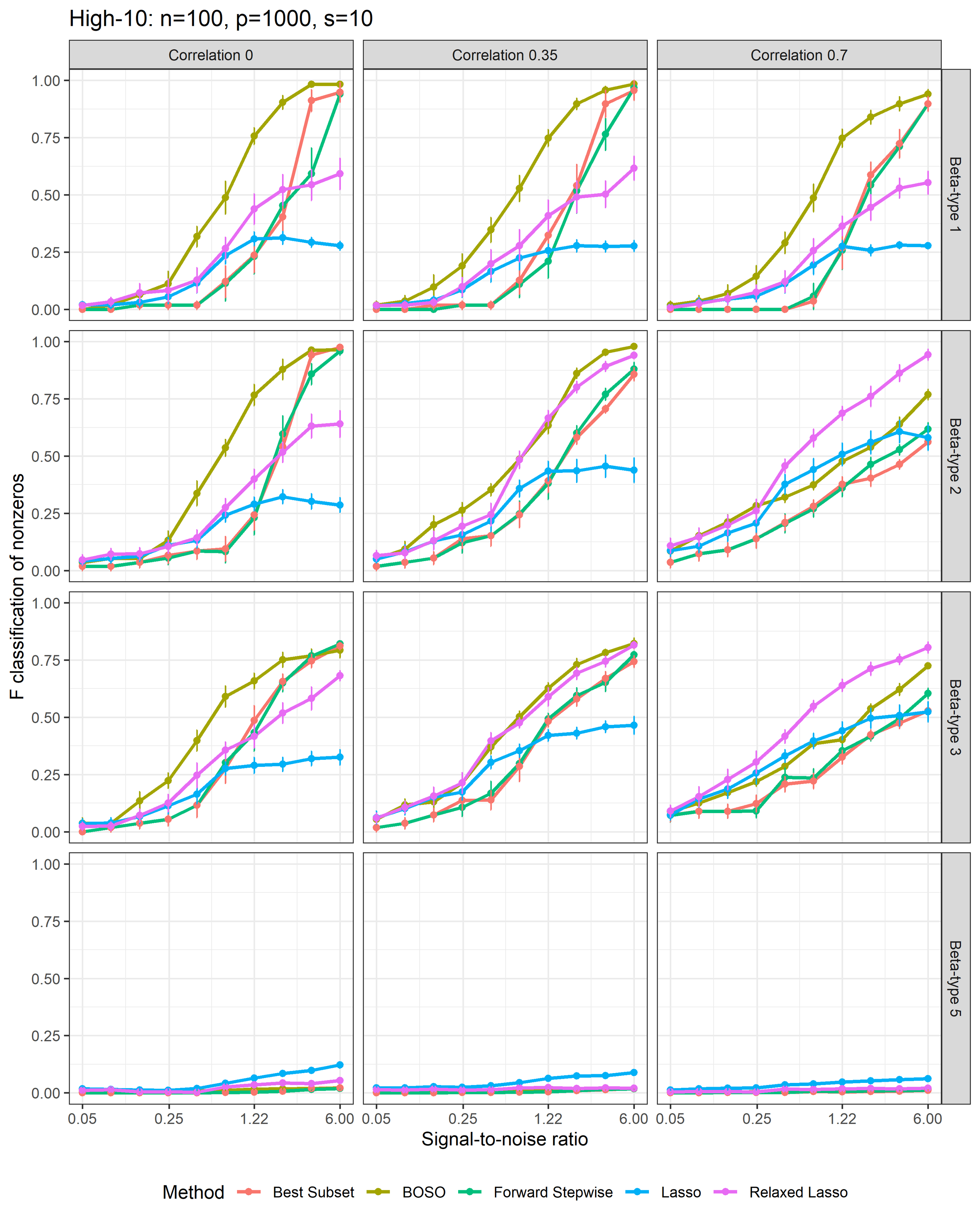

Supplement: S17 Fig — This accuracy metric is presented for the different feature selection methods (Best Subset, BOSO, Forward Stepwise, Lasso and Relaxed Lasso) and scenarios (according to Beta-type, autocorrelation levels and signal-to-noise ratio (SNR) levels) considered in the main text. S1 Appendix provides full details of the different situations considered. Points and error bars represent the mean and standard deviation in 10 random simulations, respectively. Note here that n is the number of instances, p is the total available features and s is the actual number of features contributing to the response variable. (TIF) [file pcbi.1010180.s030.tif]

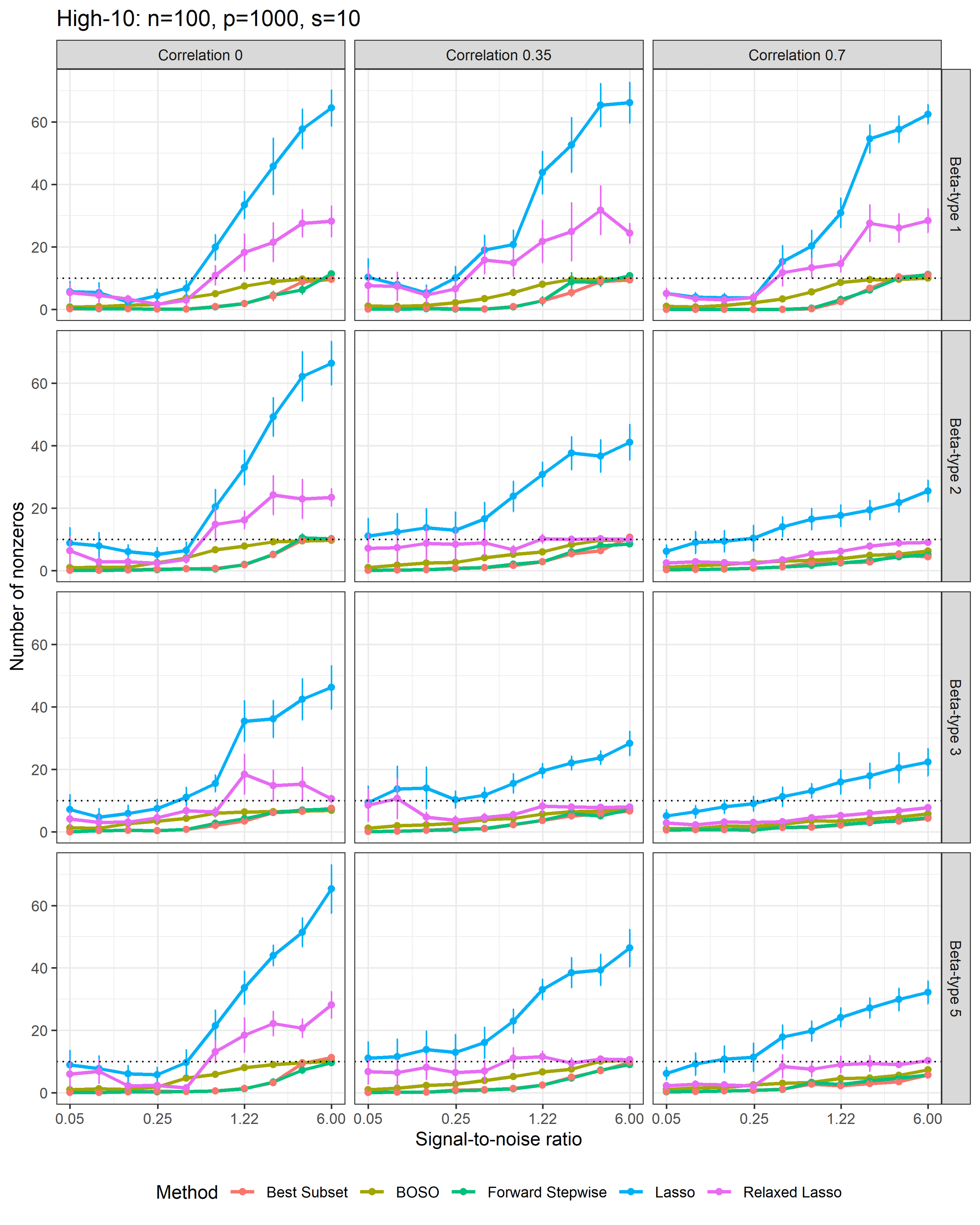

Supplement: S18 Fig — This accuracy metric is presented for the different feature selection methods (Best Subset, BOSO, Forward Stepwise, Lasso and Relaxed Lasso) and scenarios (according to Beta-type, autocorrelation levels and signal-to-noise ratio (SNR) levels) considered in the main text. S1 Appendix provides full details of the different situations considered. Points and error bars represent the mean and standard deviation in 10 random simulations, respectively. Note here that n is the number of instances, p is the total available features and s is the actual number of features contributing to the response variable. Dotted line represents the actual number of features. (TIF) [file pcbi.1010180.s031.tif]

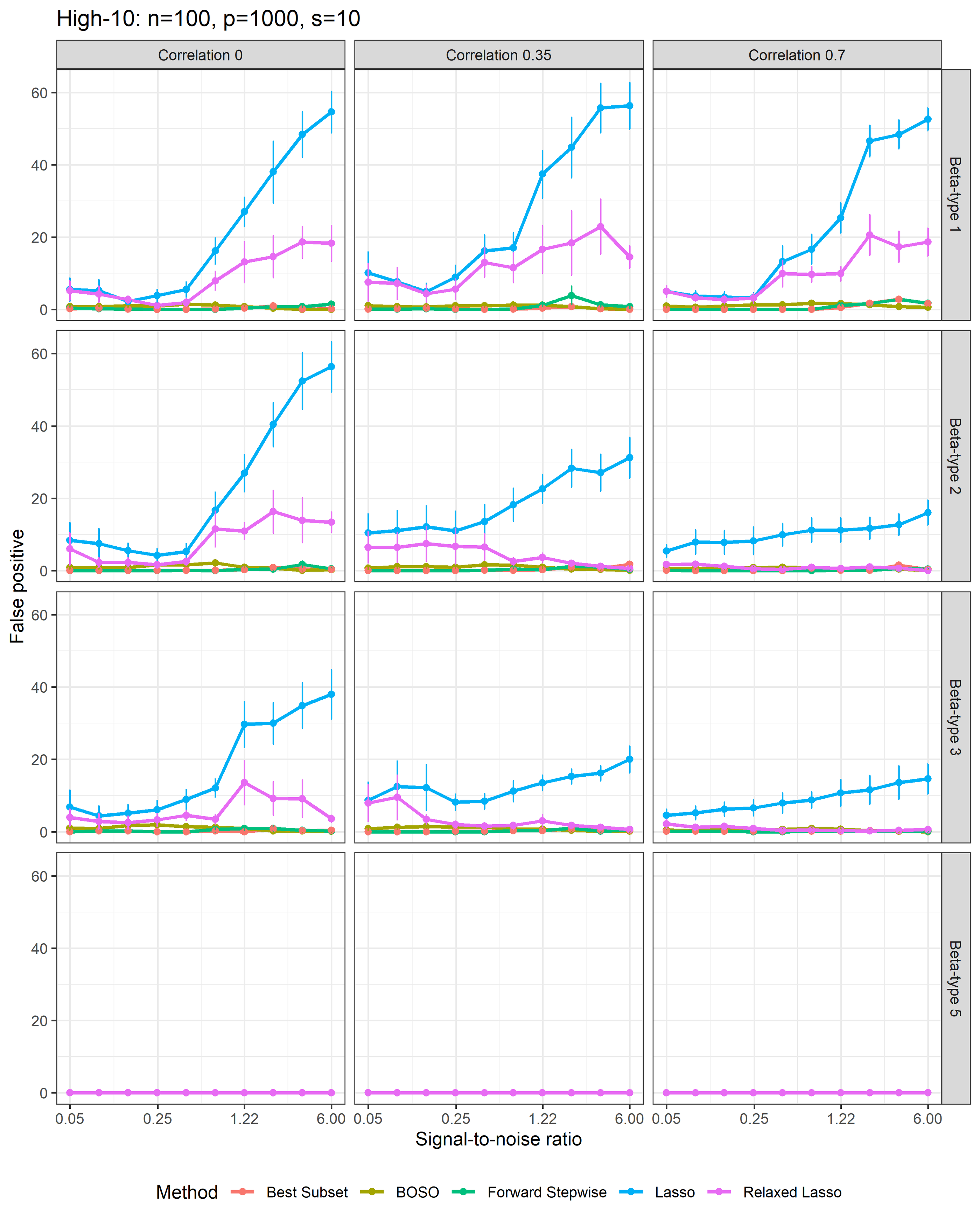

Supplement: S19 Fig — This accuracy metric is presented for the different feature selection methods (Best Subset, BOSO, Forward Stepwise, Lasso and Relaxed Lasso) and scenarios (according to Beta-type, autocorrelation levels and signal-to-noise ratio (SNR) levels) considered in the main text. S1 Appendix provides full details of the different situations considered. Points and error bars represent the mean and standard deviation in 10 random simulations, respectively. Note here that n is the number of instances, p is the total available features and s is the actual number of features contributing to the response variable. (TIF) [file pcbi.1010180.s032.tif]

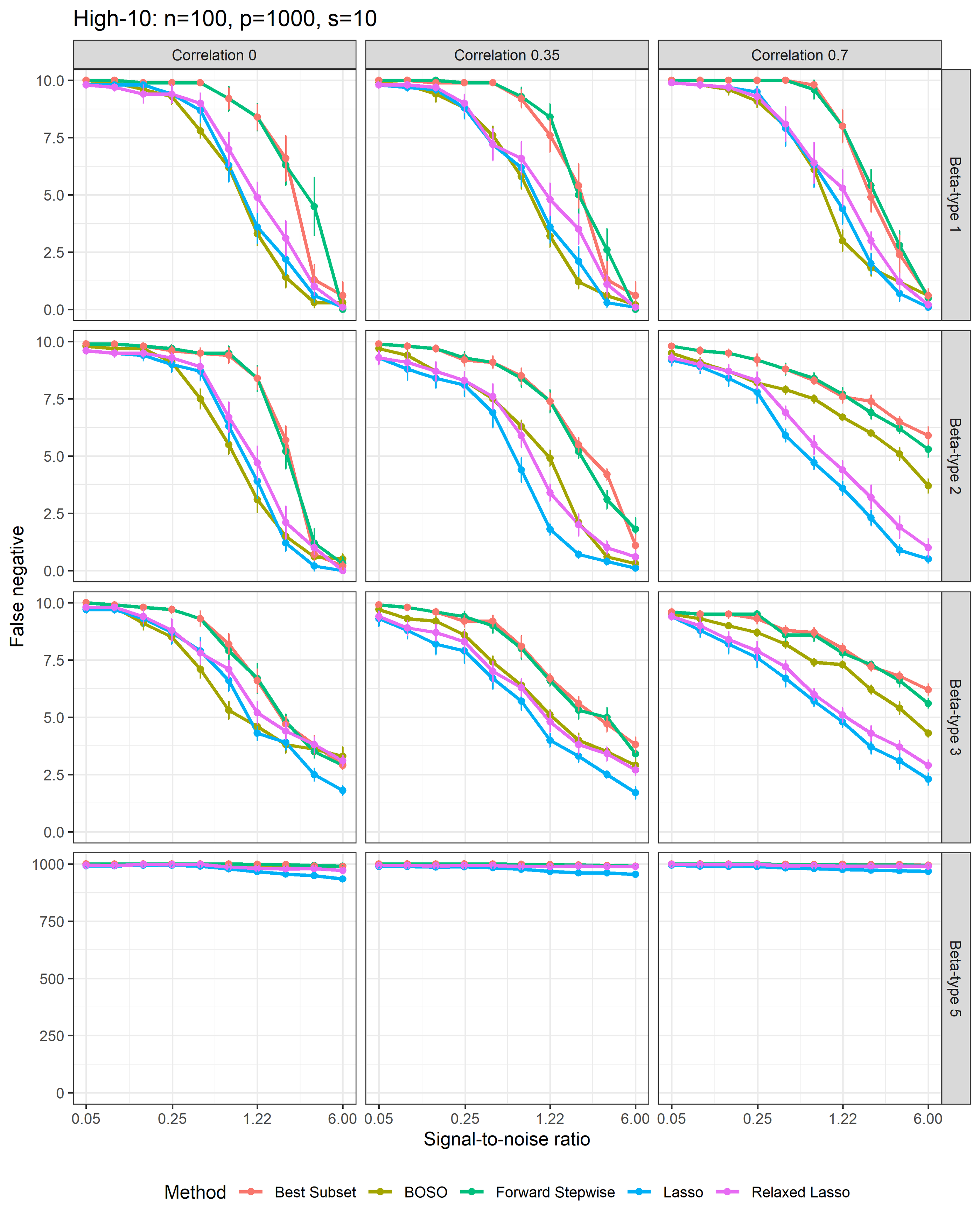

Supplement: S20 Fig — This accuracy metric is presented for the different feature selection methods (Best Subset, BOSO, Forward Stepwise, Lasso and Relaxed Lasso) and scenarios (according to Beta-type, autocorrelation levels and signal-to-noise ratio (SNR) levels) considered in the main text. S1 Appendix provides full details of the different situations considered. Points and error bars represent the mean and standard deviation in 10 random simulations, respectively. Note here that n is the number of instances, p is the total available features and s is the actual number of features contributing to the response variable. (TIF) [file pcbi.1010180.s033.tif]

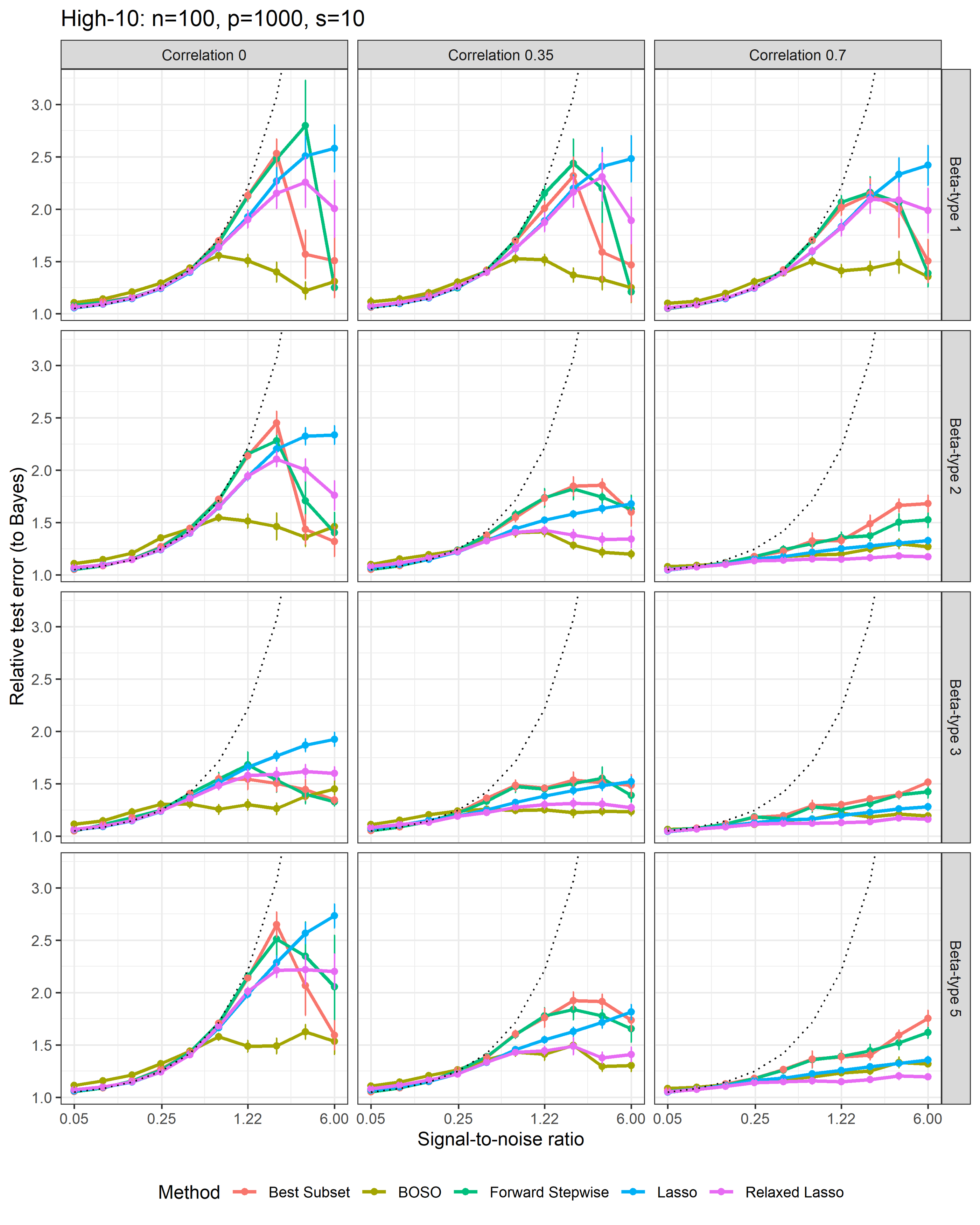

Supplement: S21 Fig — This accuracy metric is presented for the different feature selection methods (Best Subset, BOSO, Forward Stepwise, Lasso and Relaxed Lasso) and scenarios (according to Beta-type, autocorrelation levels and signal-to-noise ratio (SNR) levels) considered in the main text. S1 Appendix provides full details of the different situations considered. Points and error bars represent the mean and standard deviation in 10 random simulations, respectively. Note here that n is the number of instances, p is the total available features and s is the actual number of features contributing to the response variable. Dotted curve represents the results for the null model. (TIF) [file pcbi.1010180.s034.tif]

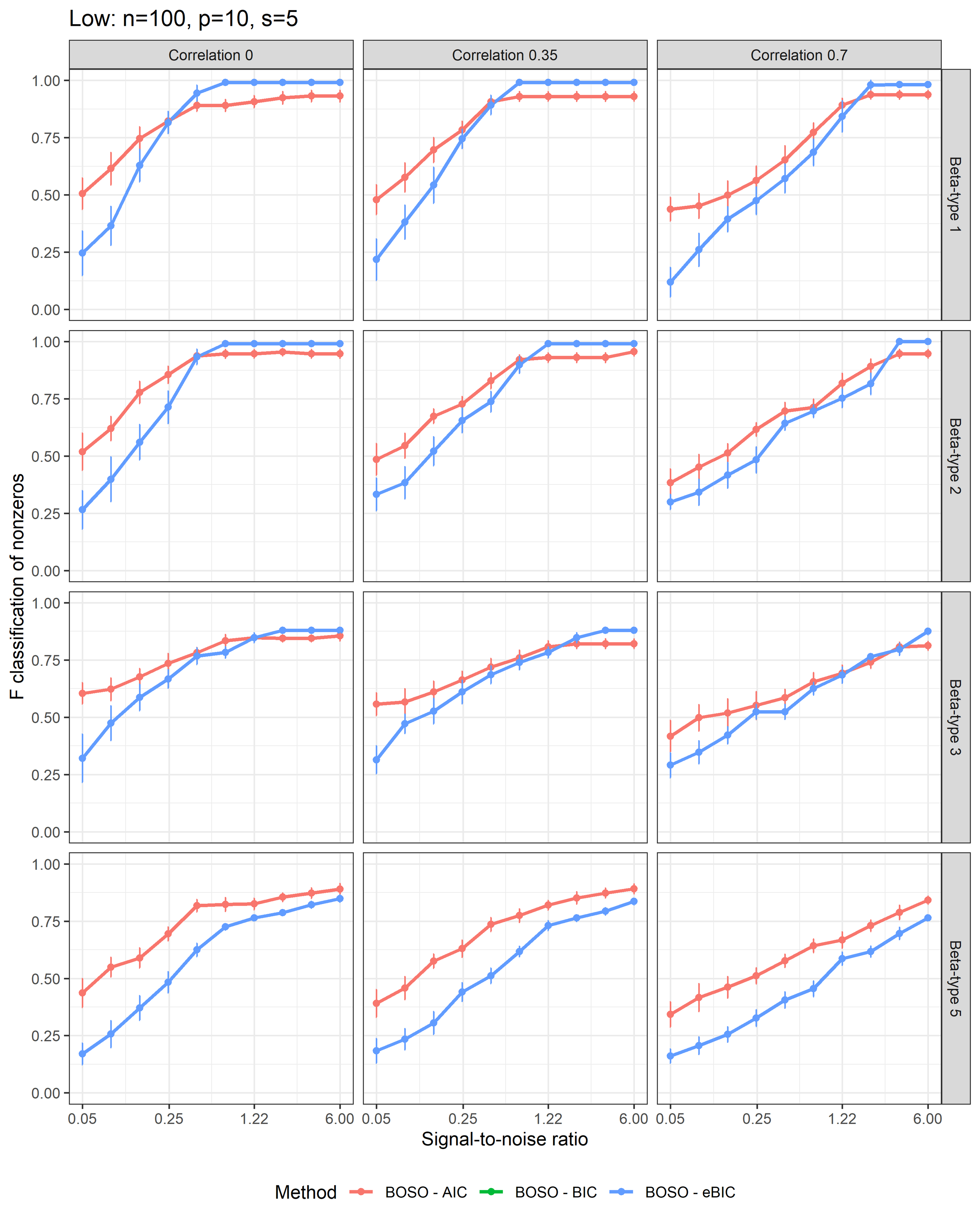

Supplement: S22 Fig — This accuracy metric is presented for BOSO under different information criteria (BOSO—AIC, BOSO—BIC and BOSO—eBIC) and scenarios (according to Beta-type, autocorrelation levels and signal-to-noise ratio (SNR) levels) considered in the main text. S1 Appendix provides full details of the different situations considered. Points and error bars represent the mean and standard deviation in 10 random simulations, respectively. Note here that n is the number of instances, p is the total available features and s is the actual number of features contributing to the response variable. (TIF) [file pcbi.1010180.s035.tif]

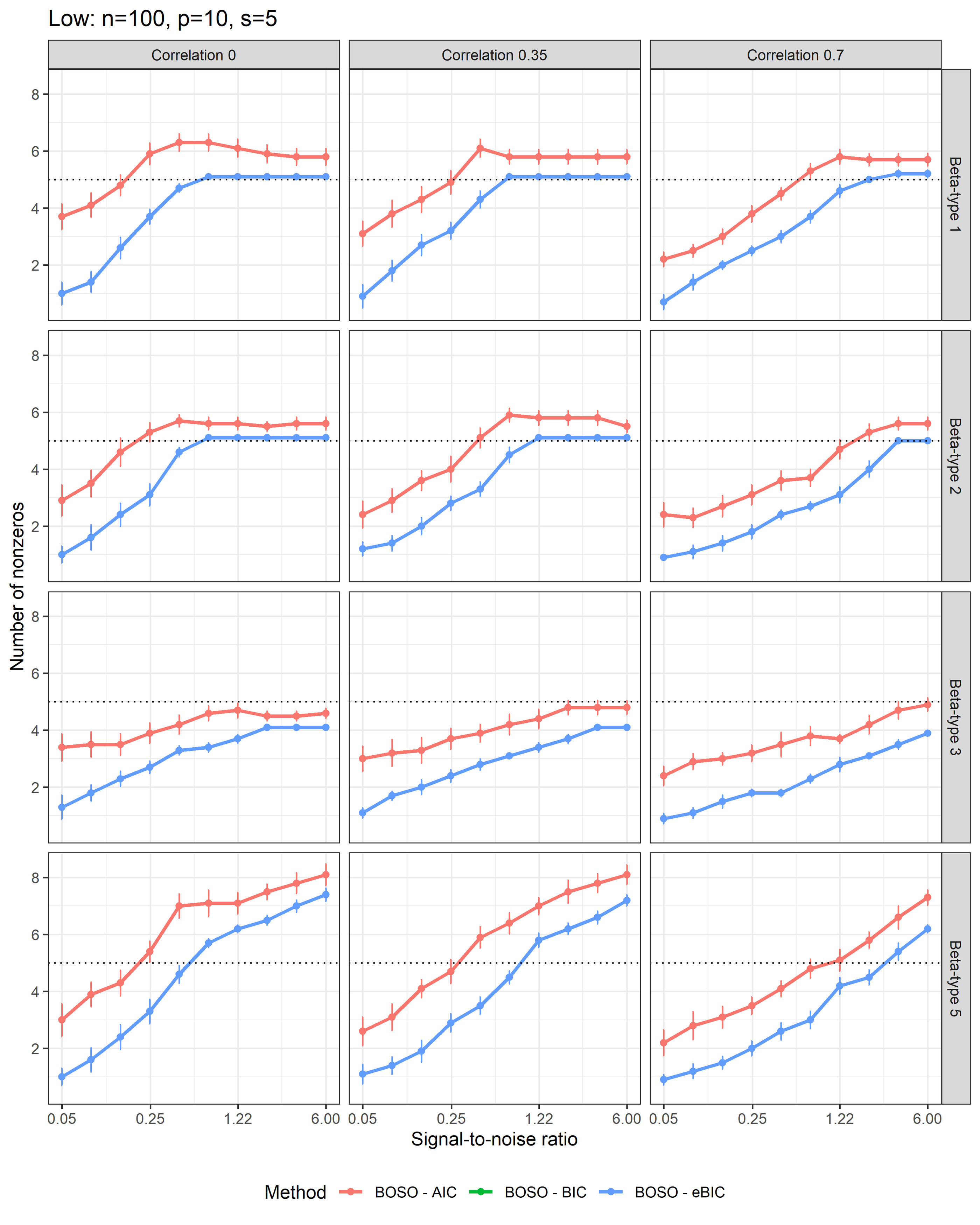

Supplement: S23 Fig — This accuracy metric is presented for BOSO under different information criteria (BOSO—AIC, BOSO—BIC and BOSO—eBIC) and scenarios (according to Beta-type, autocorrelation levels and signal-to-noise ratio (SNR) levels) considered in the main text. S1 Appendix provides full details of the different situations considered. Points and error bars represent the mean and standard deviation in 10 random simulations, respectively. Note here that n is the number of instances, p is the total available features and s is the actual number of features contributing to the response variable. The dotted line is the actual number of features. (TIF) [file pcbi.1010180.s036.tif]

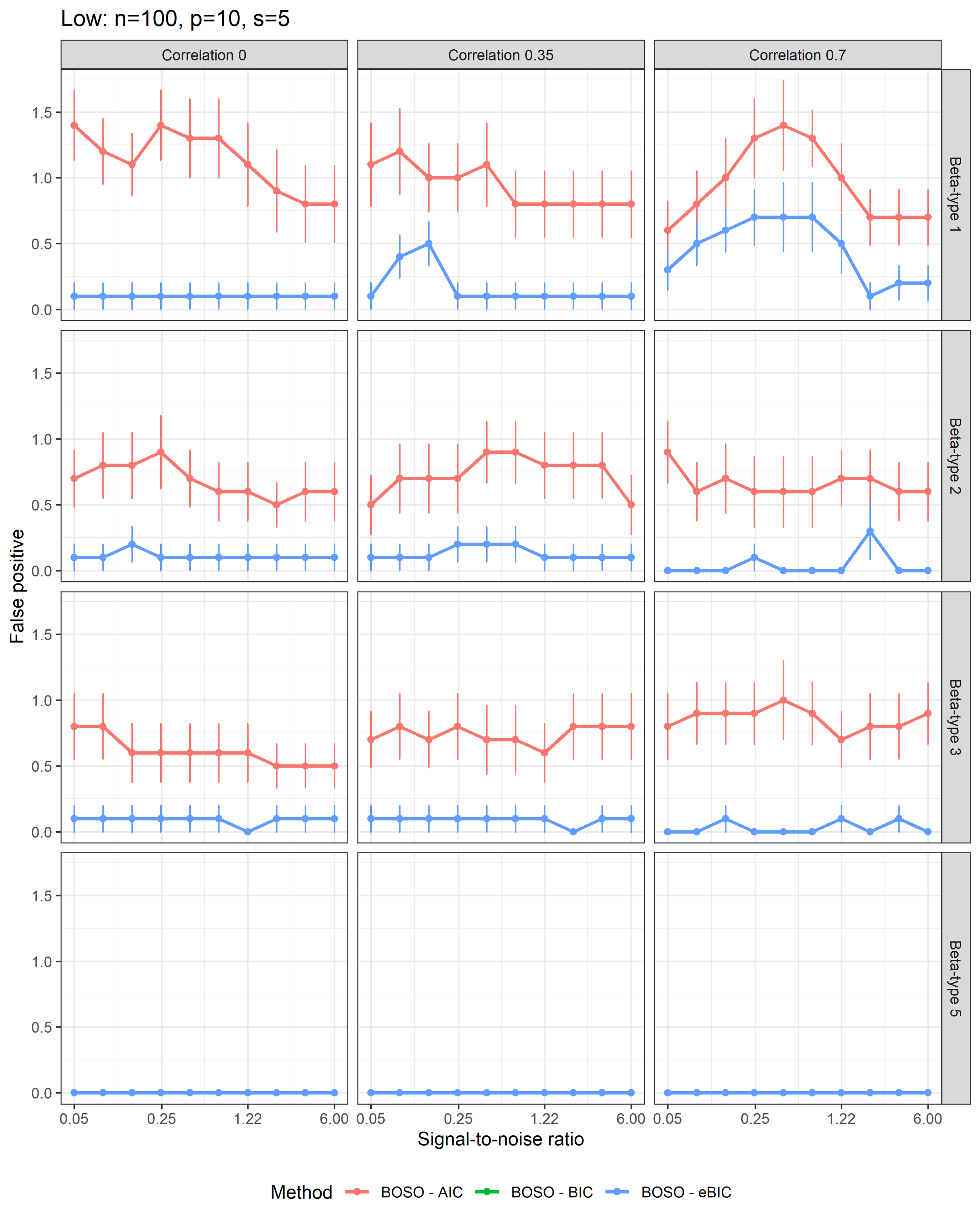

Supplement: S24 Fig — This accuracy metric is presented for BOSO under different information criteria (BOSO—AIC, BOSO—BIC and BOSO—eBIC) and scenarios (according to Beta-type, autocorrelation levels and signal-to-noise ratio (SNR) levels) considered in the main text. S1 Appendix provides full details of the different situations considered. Points and error bars represent the mean and standard deviation in 10 random simulations, respectively. Note here that n is the number of instances, p is the total available features and s is the actual number of features contributing to the response variable. (TIF) [file pcbi.1010180.s037.tif]

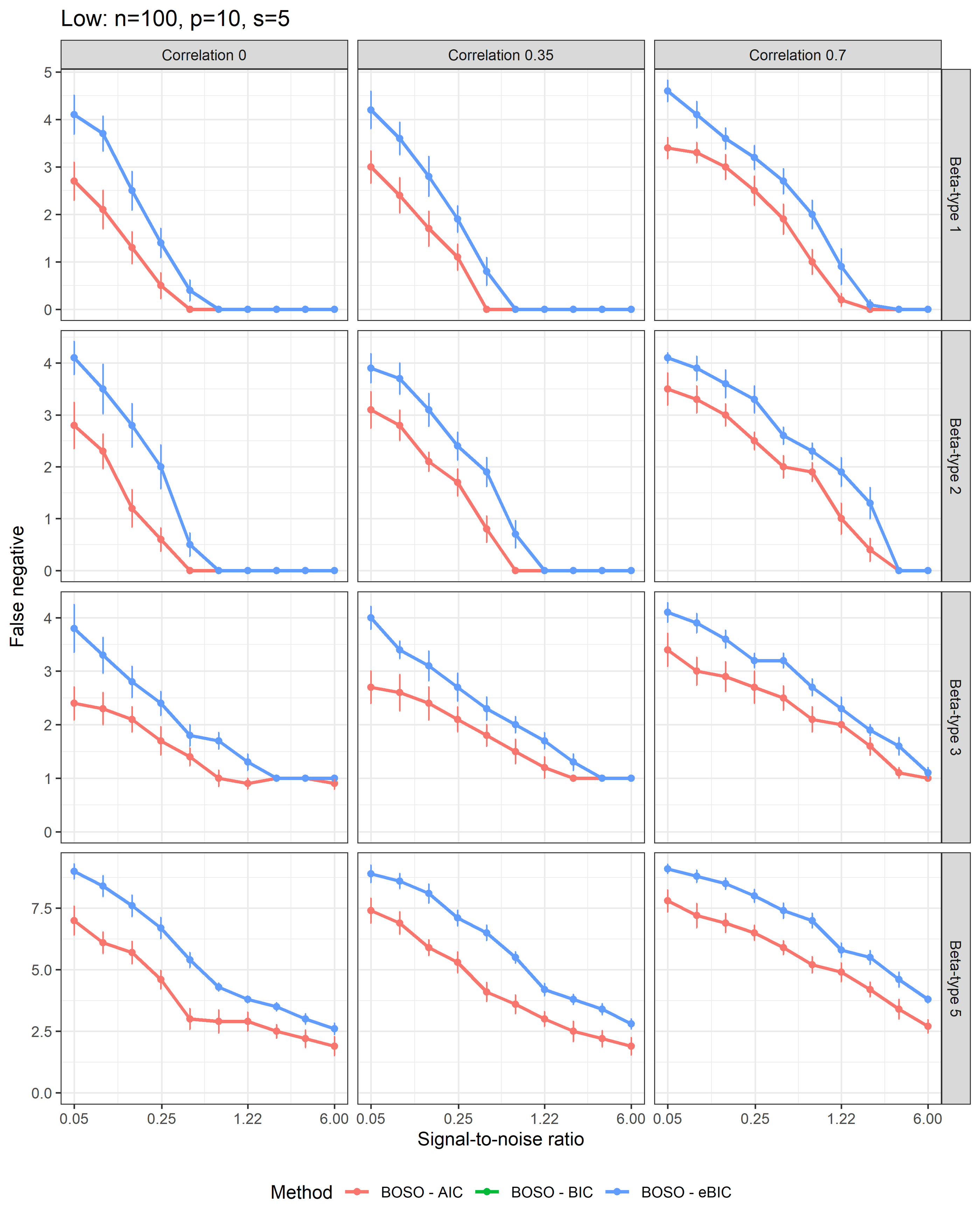

Supplement: S25 Fig — This accuracy metric is presented for BOSO under different information criteria (BOSO—AIC, BOSO—BIC and BOSO—eBIC) and scenarios (according to Beta-type, autocorrelation levels and signal-to-noise ratio (SNR) levels) considered in the main text. S1 Appendix provides full details of the different situations considered. Points and error bars represent the mean and standard deviation in 10 random simulations, respectively. Note here that n is the number of instances, p is the total available features and s is the actual number of features contributing to the response variable. (TIF) [file pcbi.1010180.s038.tif]

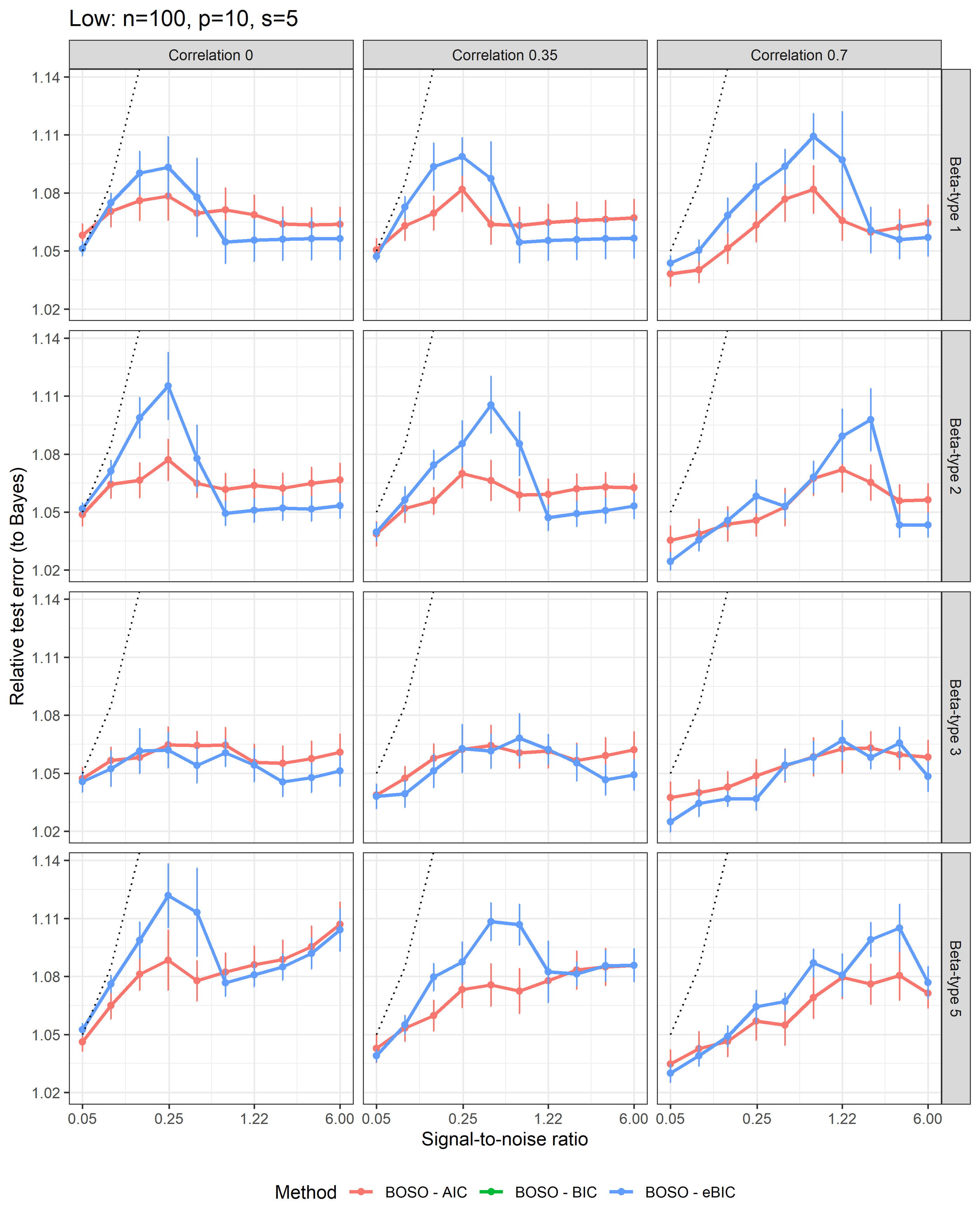

Supplement: S26 Fig — This accuracy metric is presented for BOSO under different information criteria (BOSO—AIC, BOSO—BIC and BOSO—eBIC) and scenarios (according to Beta-type, autocorrelation levels and signal-to-noise ratio (SNR) levels) considered in the main text. S1 Appendix provides full details of the different situations considered. Points and error bars represent the mean and standard deviation in 10 random simulations, respectively. Note here that n is the number of instances, p is the total available features and s is the actual number of features contributing to the response variable. Dotted curve represents the results for the null model. (TIF) [file pcbi.1010180.s039.tif]

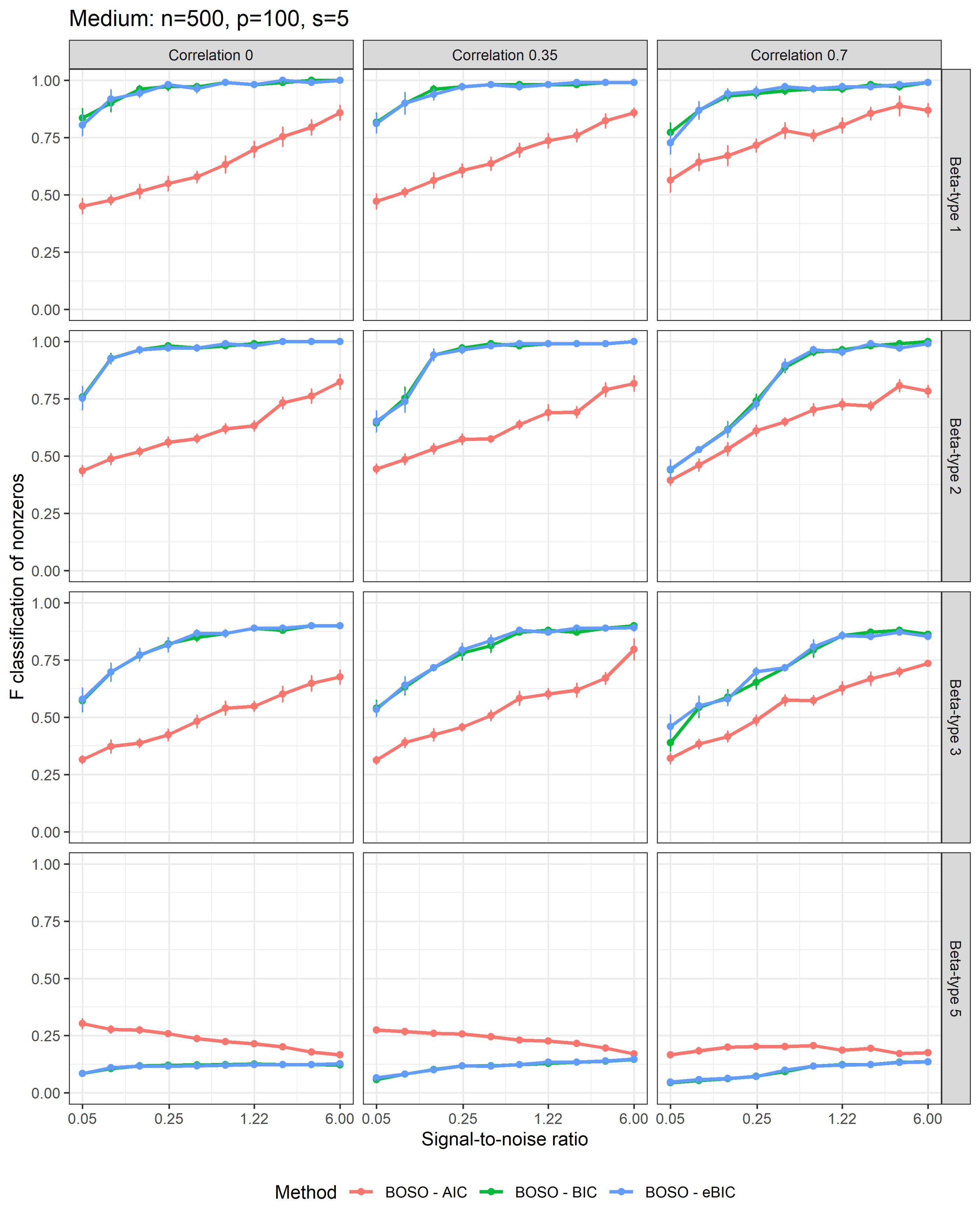

Supplement: S27 Fig — This accuracy metric is presented for BOSO under different information criteria (BOSO—AIC, BOSO—BIC and BOSO—eBIC) and scenarios (according to Beta-type, autocorrelation levels and signal-to-noise ratio (SNR) levels) considered in the main text. S1 Appendix provides full details of the different situations considered. Points and error bars represent the mean and standard deviation in 10 random simulations, respectively. Note here that n is the number of instances, p is the total available features and s is the actual number of features contributing to the response variable. (TIF) [file pcbi.1010180.s040.tif]

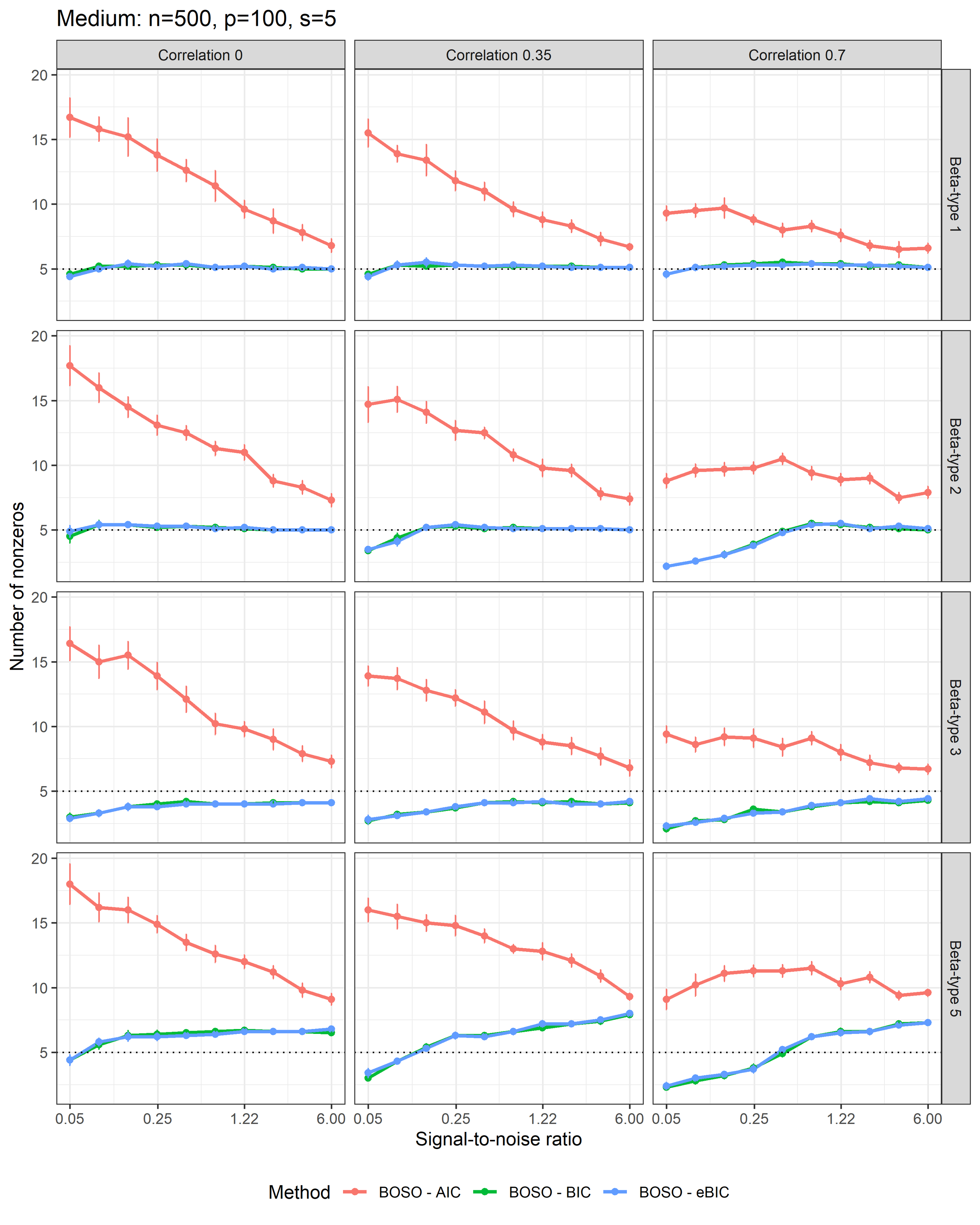

Supplement: S28 Fig — This accuracy metric is presented for BOSO under different information criteria (BOSO—AIC, BOSO—BIC and BOSO—eBIC) and scenarios (according to Beta-type, autocorrelation levels and signal-to-noise ratio (SNR) levels) considered in the main text. S1 Appendix provides full details of the different situations considered. Points and error bars represent the mean and standard deviation in 10 random simulations, respectively. Note here that n is the number of instances, p is the total available features and s is the actual number of features contributing to the response variable. Dotted line represents the actual number of features. (TIF) [file pcbi.1010180.s041.tif]

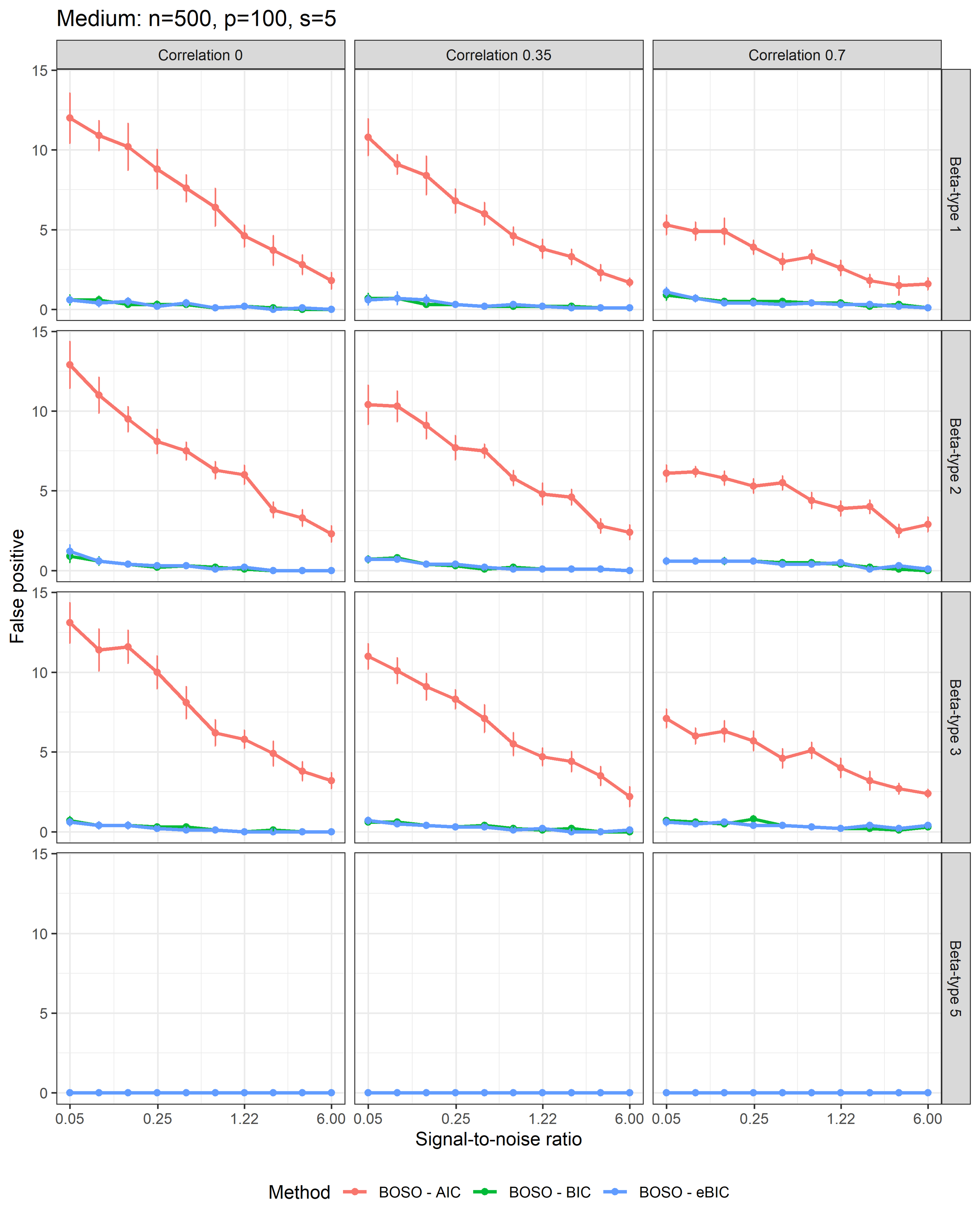

Supplement: S29 Fig — This accuracy metric is presented for BOSO under different information criteria (BOSO—AIC, BOSO—BIC and BOSO—eBIC) and scenarios (according to Beta-type, autocorrelation levels and signal-to-noise ratio (SNR) levels) considered in the main text. S1 Appendix provides full details of the different situations considered. Points and error bars represent the mean and standard deviation in 10 random simulations, respectively. Note here that n is the number of instances, p is the total available features and s is the actual number of features contributing to the response variable. (TIF) [file pcbi.1010180.s042.tif]

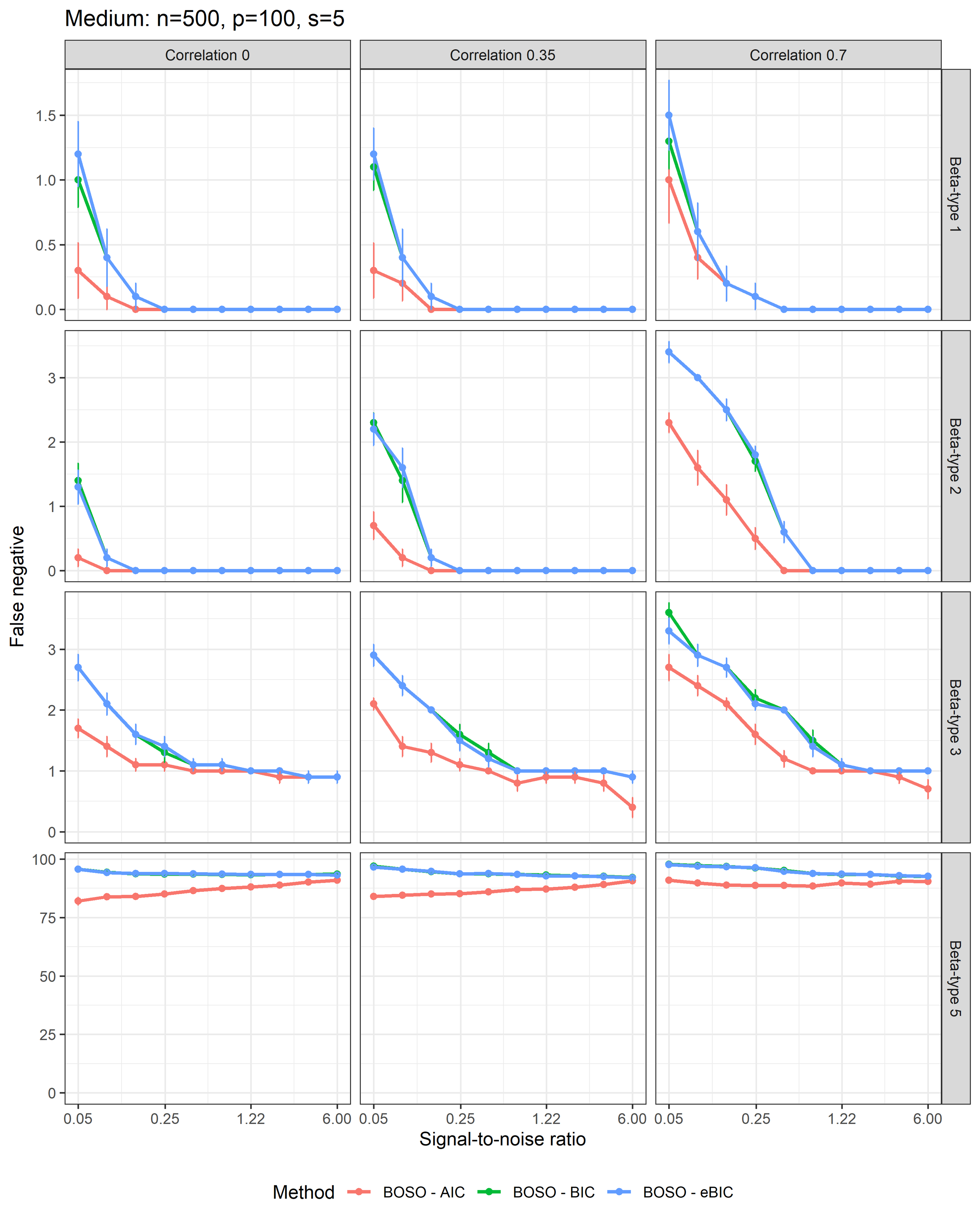

Supplement: S30 Fig — This accuracy metric is presented for BOSO under different information criteria (BOSO—AIC, BOSO—BIC and BOSO—eBIC) and scenarios (according to Beta-type, autocorrelation levels and signal-to-noise ratio (SNR) levels) considered in the main text. S1 Appendix provides full details of the different situations considered. Points and error bars represent the mean and standard deviation in 10 random simulations, respectively. Note here that n is the number of instances, p is the total available features and s is the actual number of features contributing to the response variable. (TIF) [file pcbi.1010180.s043.tif]

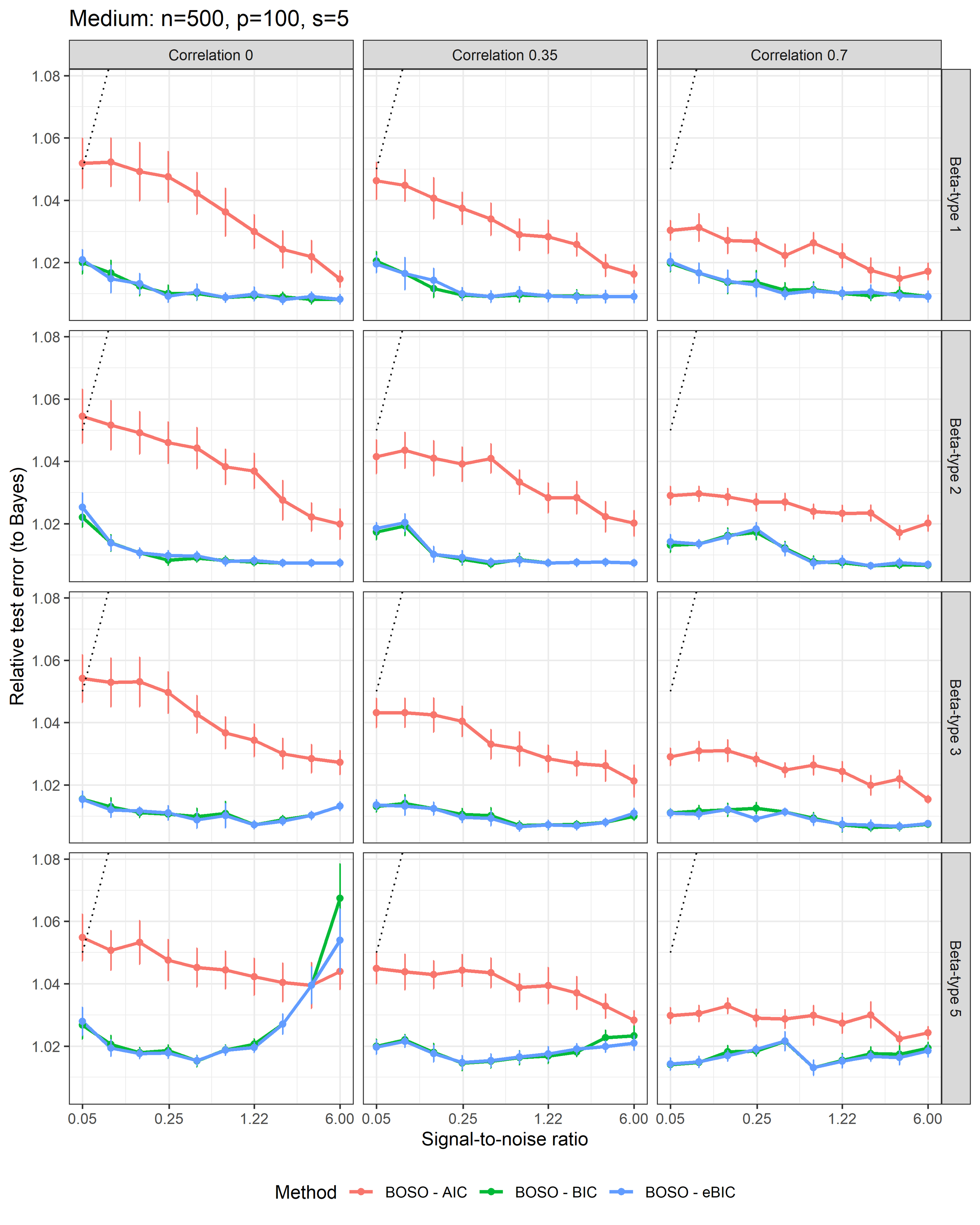

Supplement: S31 Fig — This accuracy metric is presented for BOSO under different information criteria (BOSO—AIC, BOSO—BIC and BOSO—eBIC) and scenarios (according to Beta-type, autocorrelation levels and signal-to-noise ratio (SNR) levels) considered in the main text. S1 Appendix provides full details of the different situations considered. Points and error bars represent the mean and standard deviation in 10 random simulations, respectively. Note here that n is the number of instances, p is the total available features and s is the actual number of features contributing to the response variable. Dotted line represents the results for the null model. (TIF) [file pcbi.1010180.s044.tif]

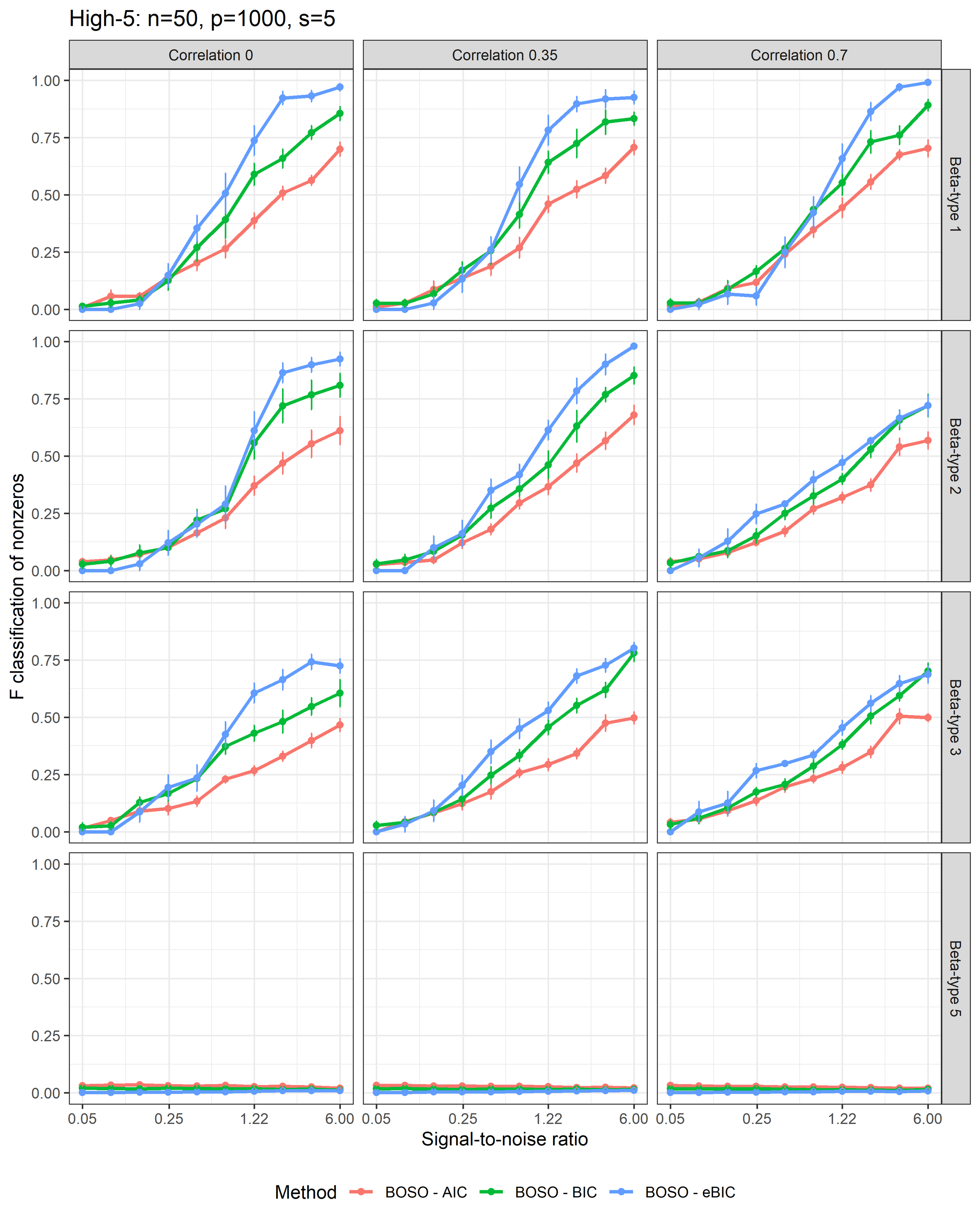

Supplement: S32 Fig — This accuracy metric is presented for BOSO under different information criteria (BOSO—AIC, BOSO—BIC and BOSO—eBIC) and scenarios (according to Beta-type, autocorrelation levels and signal-to-noise ratio (SNR) levels) considered in the main text. S1 Appendix provides full details of the different situations considered. Points and error bars represent the mean and standard deviation in 10 random simulations, respectively. Note here that n is the number of instances, p is the total available features and s is the actual number of features contributing to the response variable. (TIF) [file pcbi.1010180.s045.tif]

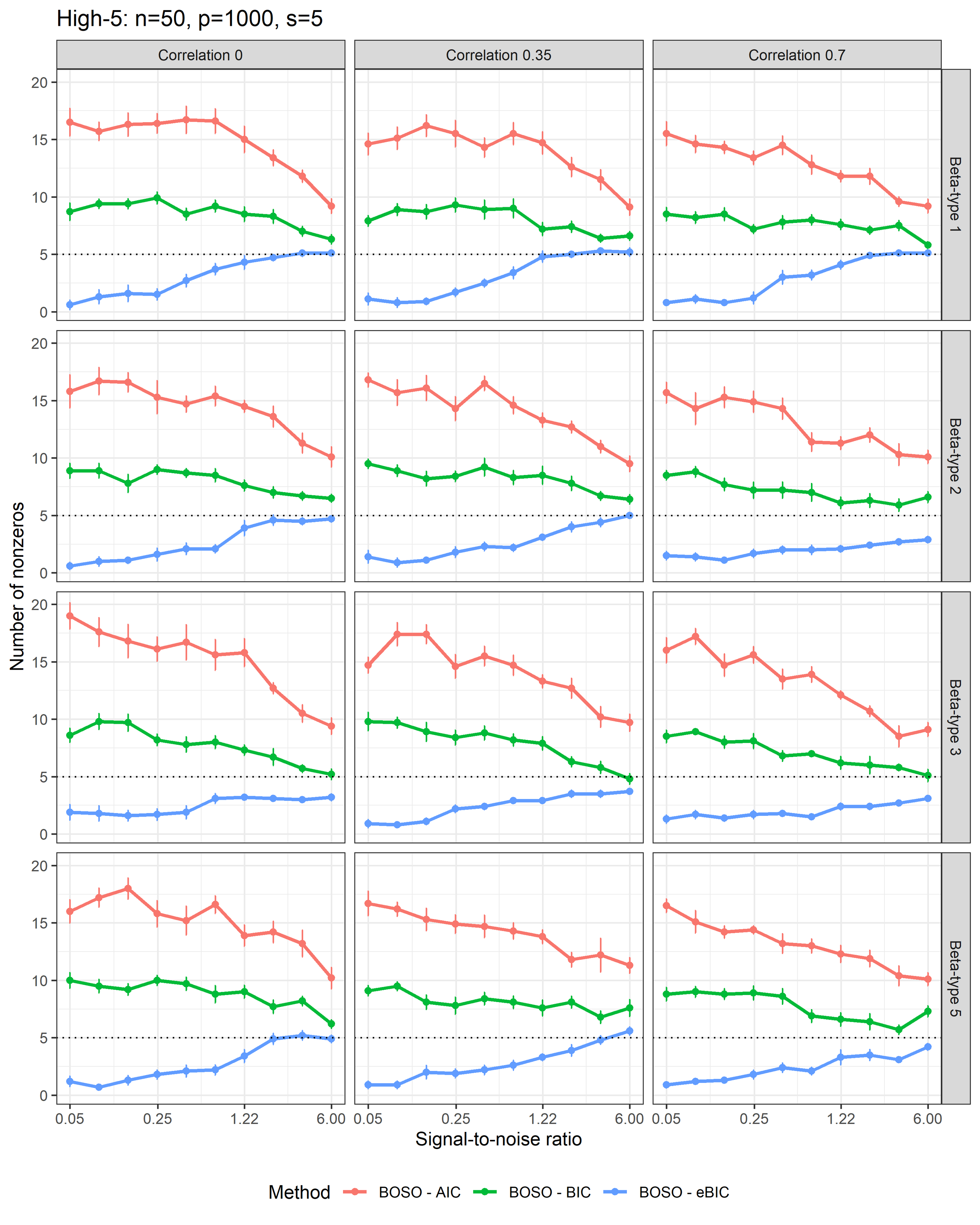

Supplement: S33 Fig — This accuracy metric is presented for BOSO under different information criteria (BOSO—AIC, BOSO—BIC and BOSO—eBIC) and scenarios (according to Beta-type, autocorrelation levels and signal-to-noise ratio (SNR) levels) considered in the main text. S1 Appendix provides full details of the different situations considered. Points and error bars represent the mean and standard deviation in 10 random simulations, respectively. Note here that n is the number of instances, p is the total available features and s is the actual number of features contributing to the response variable. The dotted line represents the actual number of features. (TIF) [file pcbi.1010180.s046.tif]

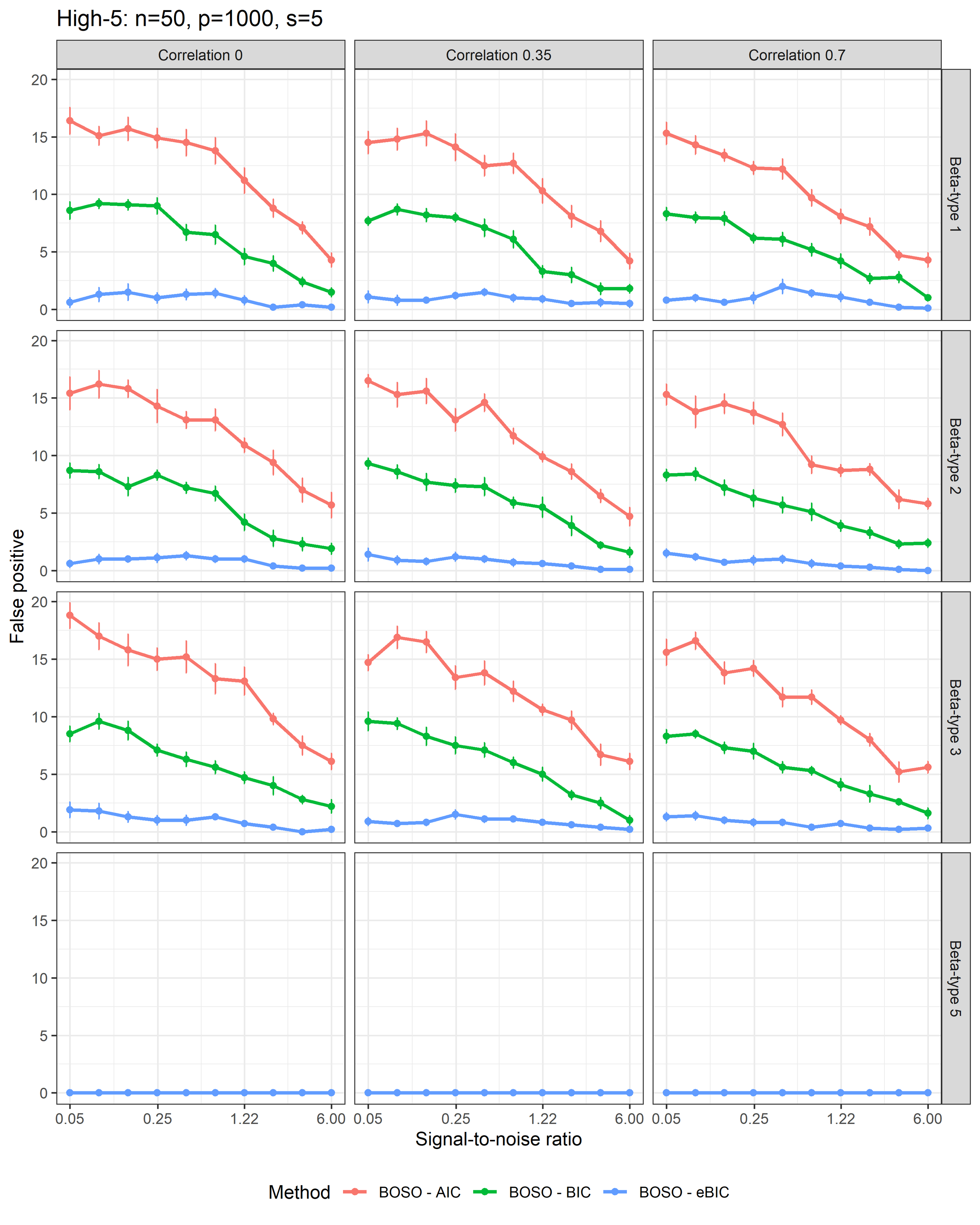

Supplement: S34 Fig — This accuracy metric is presented for BOSO under different information criteria (BOSO—AIC, BOSO—BIC and BOSO—eBIC) and scenarios (according to Beta-type, autocorrelation levels and signal-to-noise ratio (SNR) levels) considered in the main text. S1 Appendix provides full details of the different situations considered. Points and error bars represent the mean and standard deviation in 10 random simulations, respectively. Note here that n is the number of instances, p is the total available features and s is the actual number of features contributing to the response variable. (TIF) [file pcbi.1010180.s047.tif]

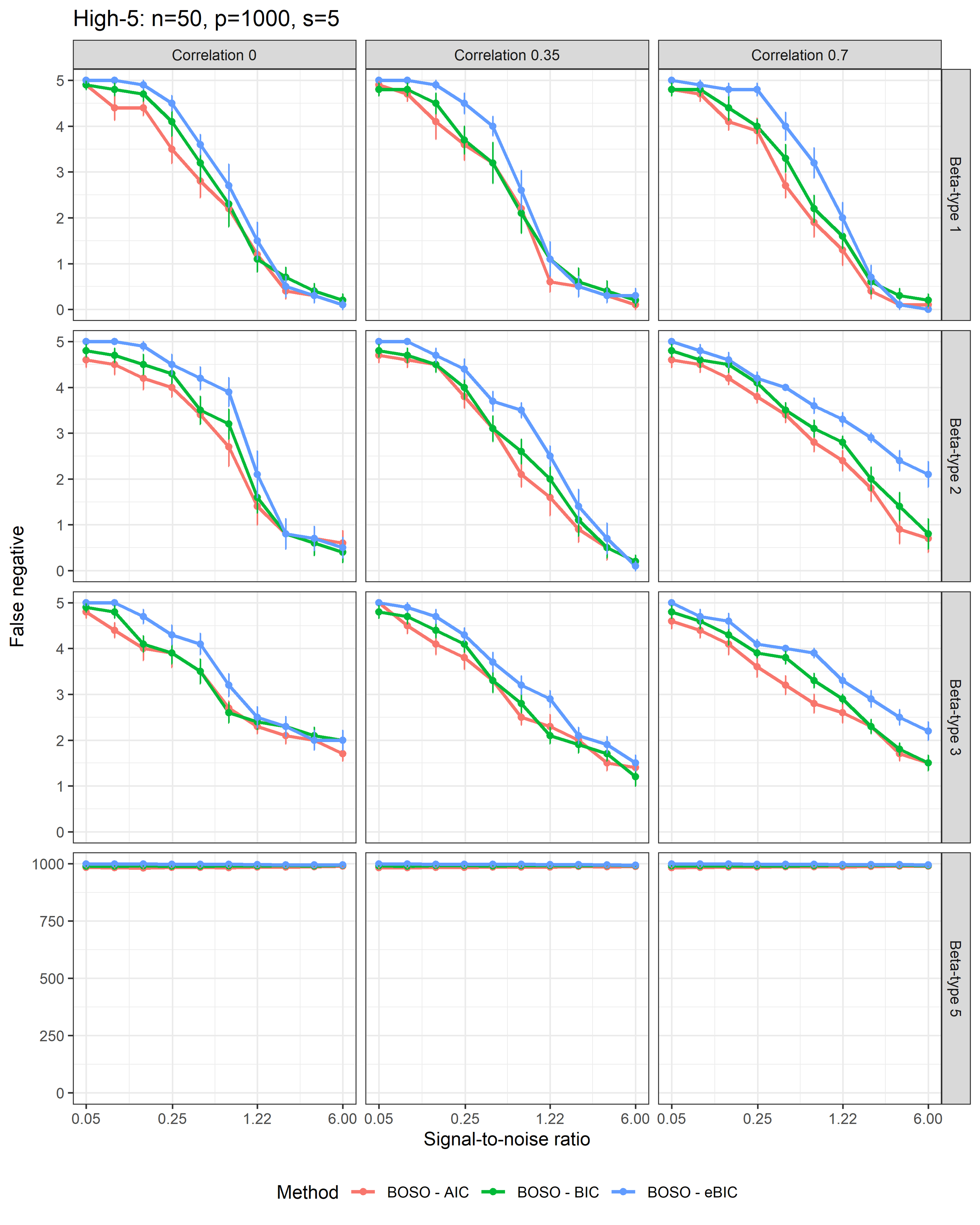

Supplement: S35 Fig — This accuracy metric is presented for BOSO under different information criteria (BOSO—AIC, BOSO—BIC and BOSO—eBIC) and scenarios (according to Beta-type, autocorrelation levels and signal-to-noise ratio (SNR) levels) considered in the main text. S1 Appendix provides full details of the different situations considered. Points and error bars represent the mean and standard deviation in 10 random simulations, respectively. Note here that n is the number of instances, p is the total available features and s is the actual number of features contributing to the response variable. (TIF) [file pcbi.1010180.s048.tif]

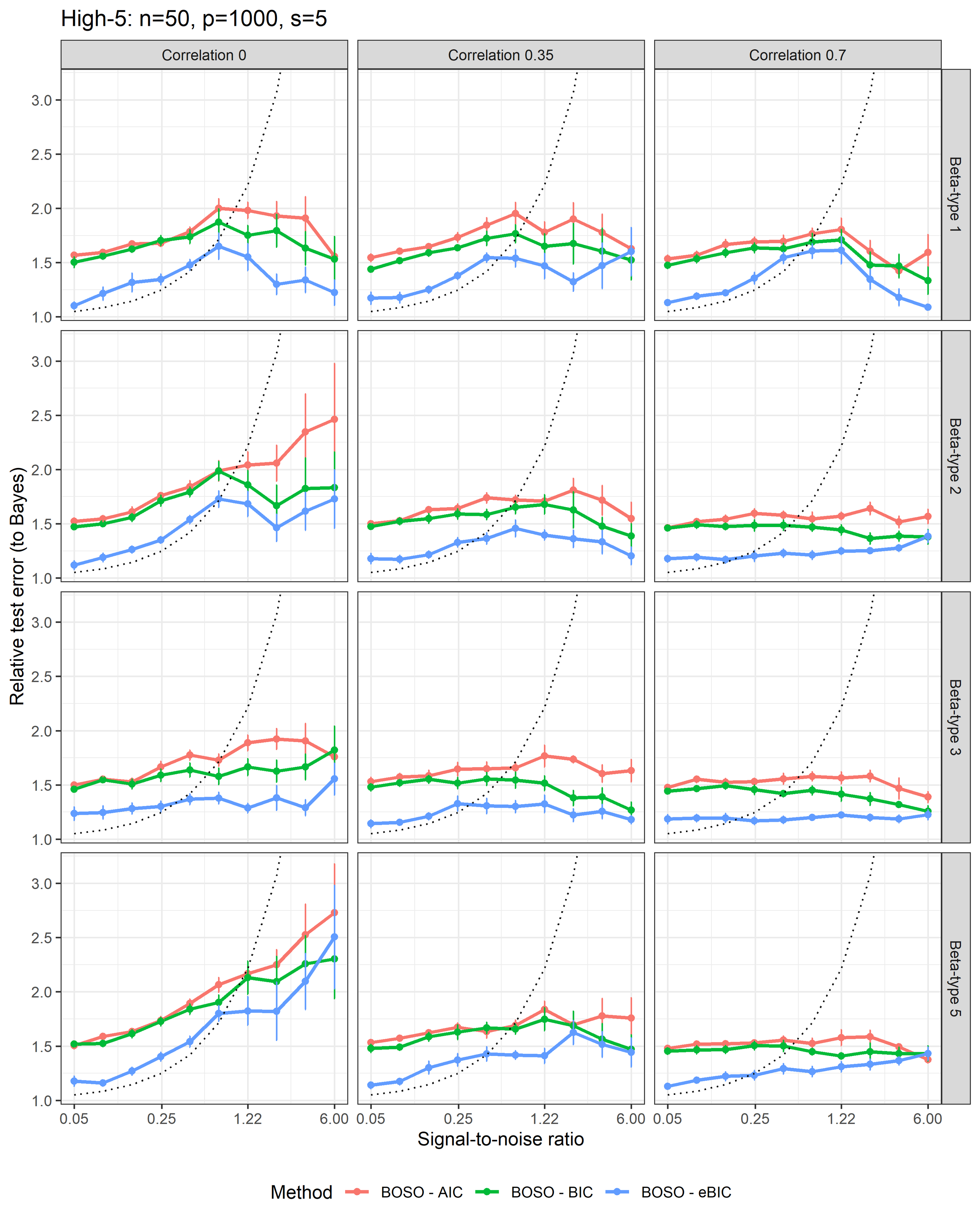

Supplement: S36 Fig — This accuracy metric is presented for BOSO under different information criteria (BOSO—AIC, BOSO—BIC and BOSO—eBIC) and scenarios (according to Beta-type, autocorrelation levels and signal-to-noise ratio (SNR) levels) considered in the main text. S1 Appendix provides full details of the different situations considered. Points and error bars represent the mean and standard deviation in 10 random simulations, respectively. Note here that n is the number of instances, p is the total available features and s is the actual number of features contributing to the response variable. Dotted curve represents the results for the null model. (TIF) [file pcbi.1010180.s049.tif]

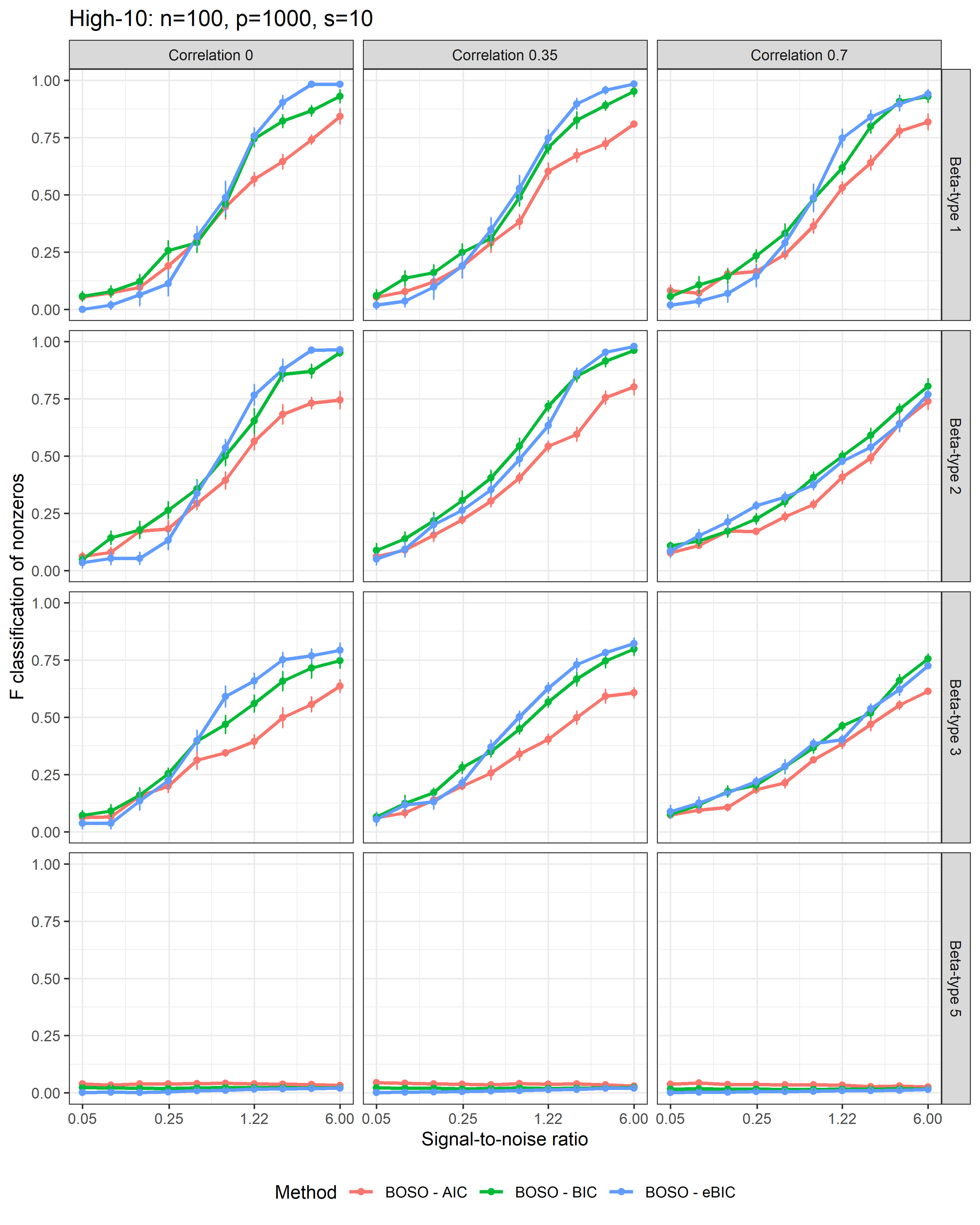

Supplement: S37 Fig — This accuracy metric is presented for BOSO under different information criteria (BOSO—AIC, BOSO—BIC and BOSO—eBIC) and scenarios (according to Beta-type, autocorrelation levels and signal-to-noise ratio (SNR) levels) considered in the main text. S1 Appendix provides full details of the different situations considered. Points and error bars represent the mean and standard deviation in 10 random simulations, respectively. Note here that n is the number of instances, p is the total available features and s is the actual number of features contributing to the response variable. (TIF) [file pcbi.1010180.s050.tif]

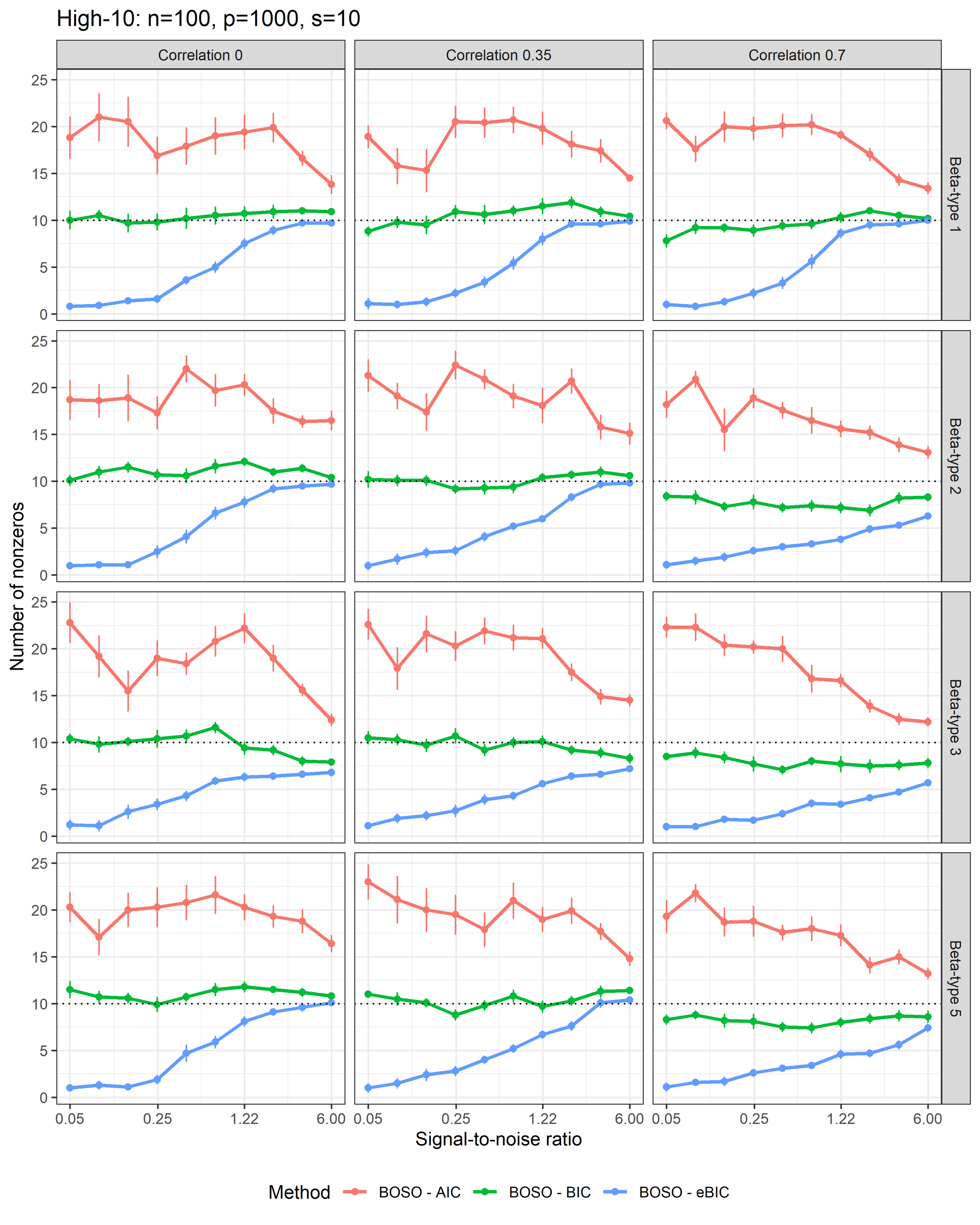

Supplement: S38 Fig — This accuracy metric is presented for BOSO under different information criteria (BOSO—AIC, BOSO—BIC and BOSO—eBIC) and scenarios (according to Beta-type, autocorrelation levels and signal-to-noise ratio (SNR) levels) considered in the main text. S1 Appendix provides full details of the different situations considered. Points and error bars represent the mean and standard deviation in 10 random simulations, respectively. Note here that n is the number of instances, p is the total available features and s is the actual number of features contributing to the response variable. The dotted line represents the actual number of features. (TIF) [file pcbi.1010180.s051.tif]

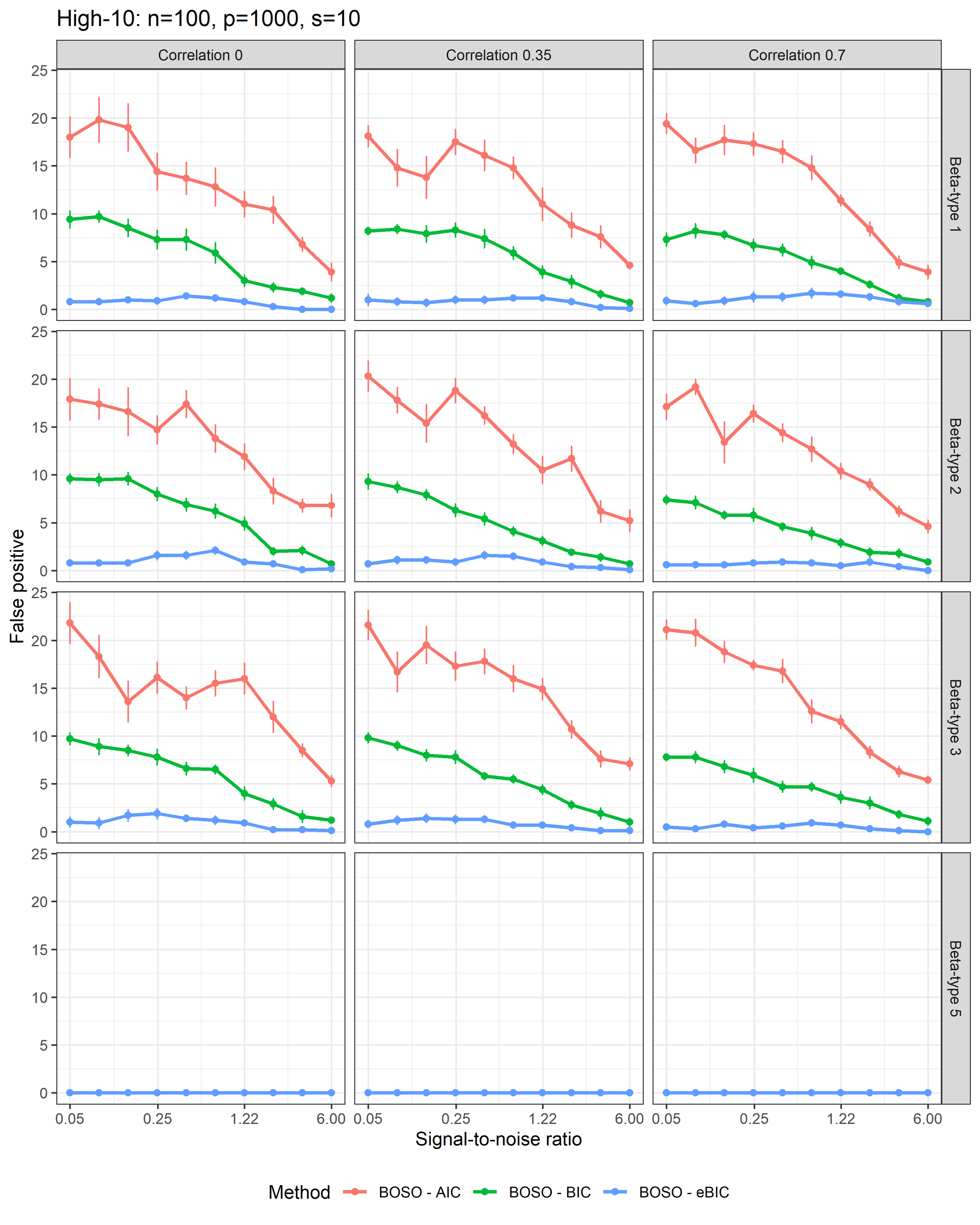

Supplement: S39 Fig — This accuracy metric is presented for BOSO under different information criteria (BOSO—AIC, BOSO—BIC and BOSO—eBIC) and scenarios (according to Beta-type, autocorrelation levels and signal-to-noise ratio (SNR) levels) considered in the main text. S1 Appendix provides full details of the different situations considered. Points and error bars represent the mean and standard deviation in 10 random simulations, respectively. Note here that n is the number of instances, p is the total available features and s is the actual number of features contributing to the response variable. (TIF) [file pcbi.1010180.s052.tif]

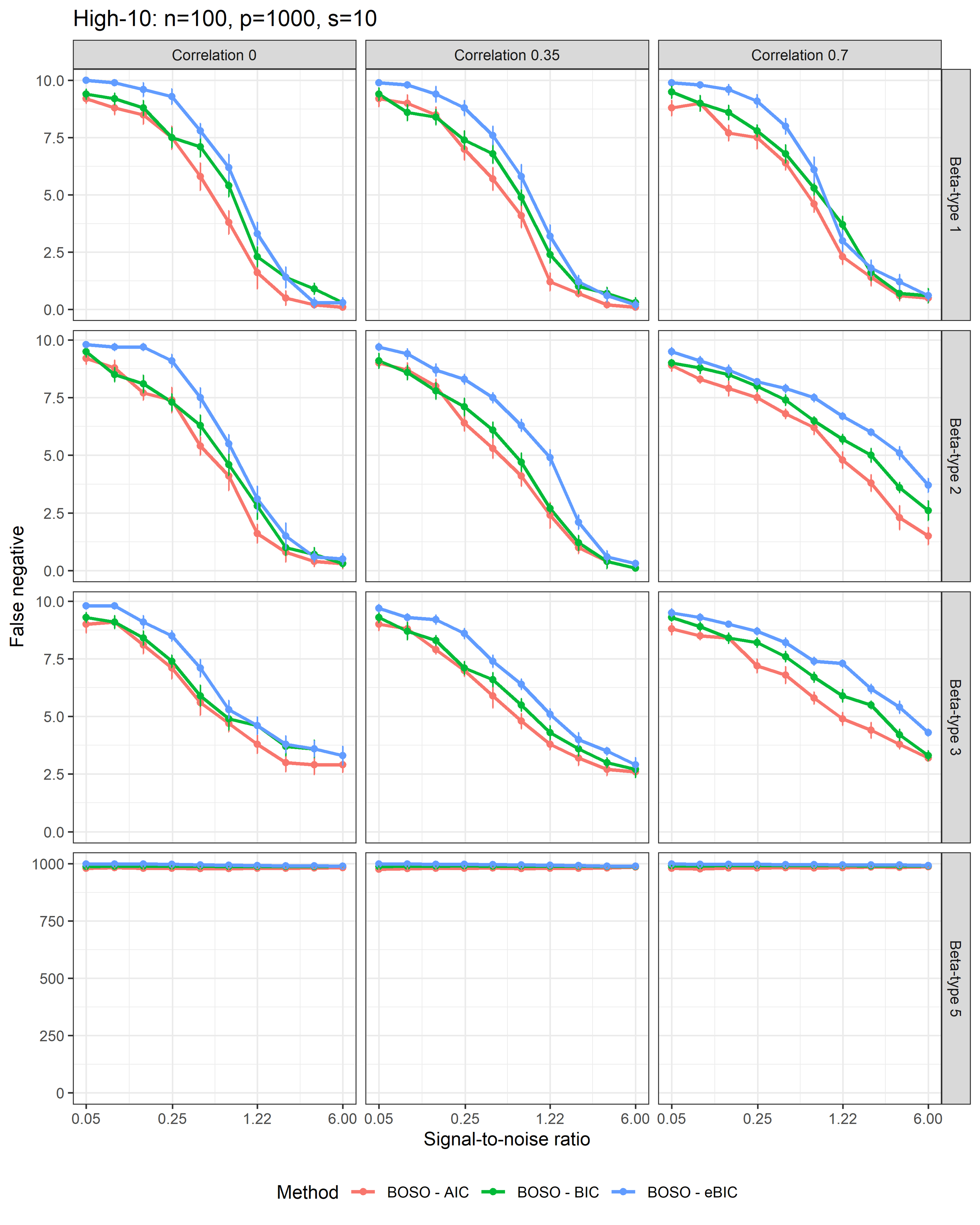

Supplement: S40 Fig — This accuracy metric is presented for BOSO under different information criteria (BOSO—AIC, BOSO—BIC and BOSO—eBIC) and scenarios (according to Beta-type, autocorrelation levels and signal-to-noise ratio (SNR) levels) considered in the main text. S1 Appendix provides full details of the different situations considered. Points and error bars represent the mean and standard deviation in 10 random simulations, respectively. Note here that n is the number of instances, p is the total available features and s is the actual number of features contributing to the response variable. (TIF) [file pcbi.1010180.s053.tif]

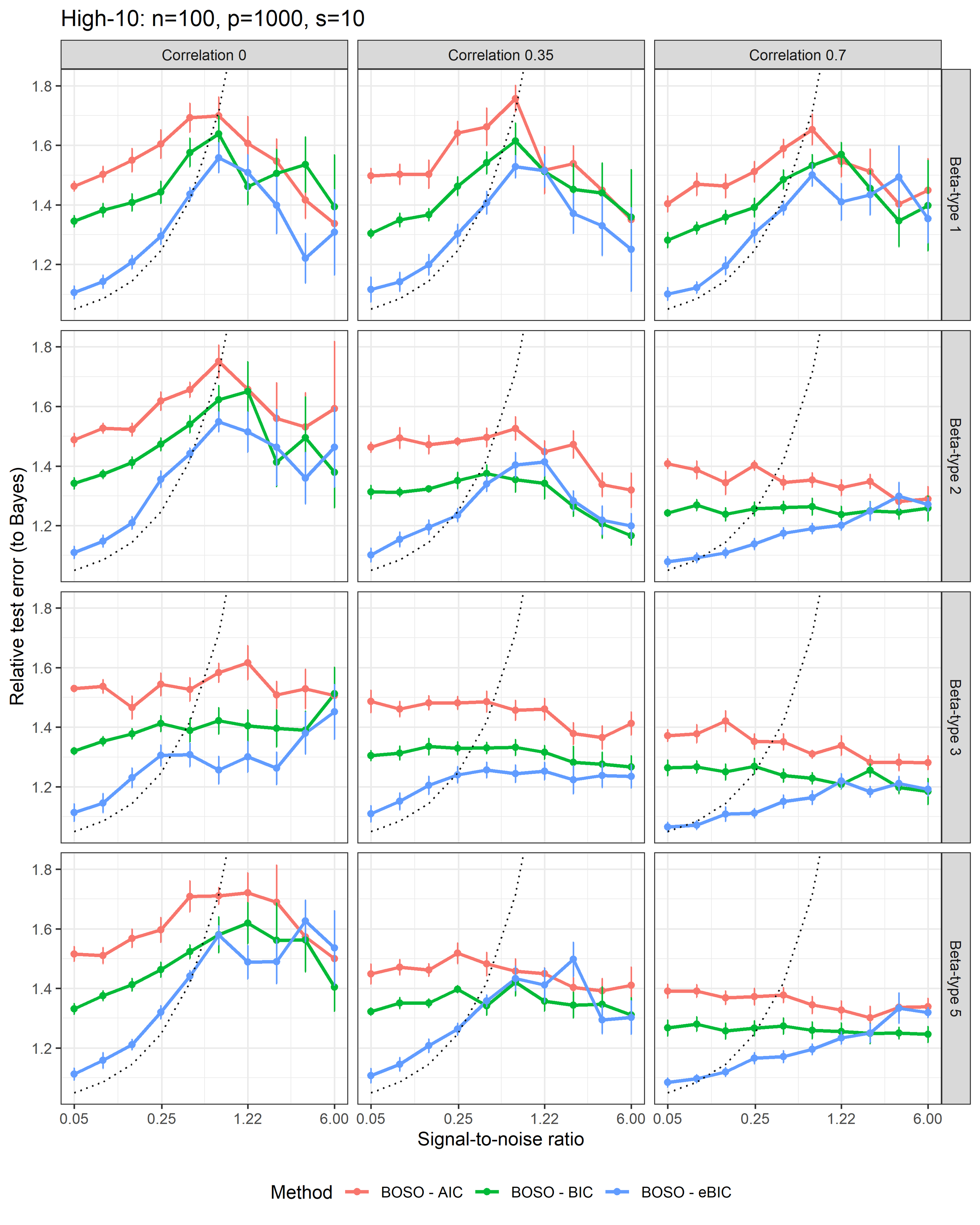

Supplement: S41 Fig — This accuracy metric is presented for BOSO under different information criteria (BOSO—AIC, BOSO—BIC and BOSO—eBIC) and scenarios (according to Beta-type, autocorrelation levels and signal-to-noise ratio (SNR) levels) considered in the main text. S1 Appendix provides full details of the different situations considered. Points and error bars represent the mean and standard deviation in 10 random simulations, respectively. Note here that n is the number of instances, p is the total available features and s is the actual number of features contributing to the response variable. Dotted curve represents the results for the null model. (TIF) [file pcbi.1010180.s054.tif]

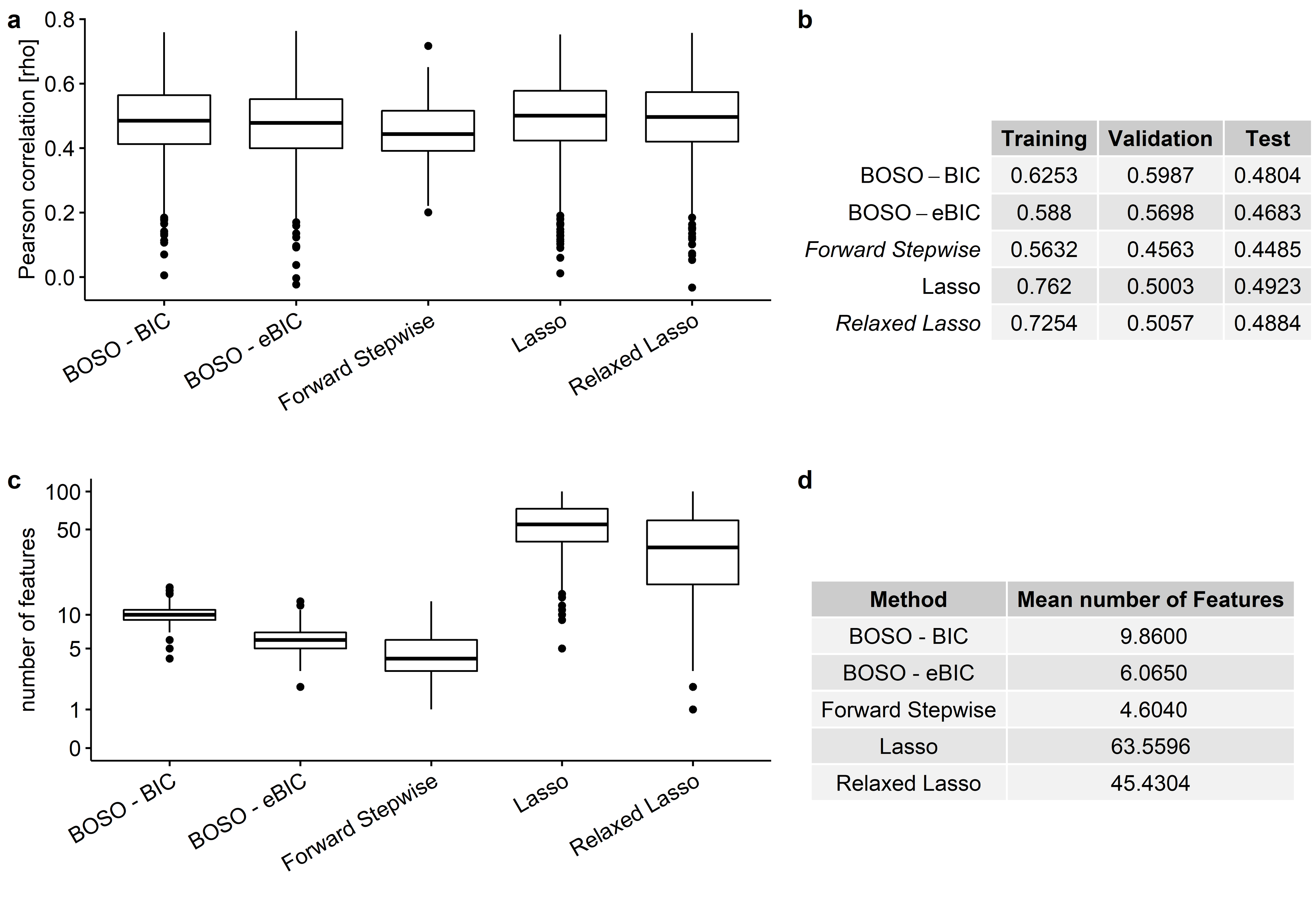

Supplement: S42 Fig — a) For 20 random partitions into training, validation and test data of the 50 drugs detailed in S8 Table, comparison of the Pearson Correlation values between GDSC IC50 and predicted IC50 values with BOSO-BIC, BOSO-eBIC, Forward Stepwise, Lasso and Relaxed Lasso, respectively, in the Test partition; b) Summary table of mean Pearson Correlation values for the analyzed cases in ‘a’ panel in the three data partitions; c) Comparison of number of active features for the analyzed cases in ‘a’; d) Summary table for the mean number of selected variables for the analyzed cases in ‘a’. (TIF) [file pcbi.1010180.s055.tif]

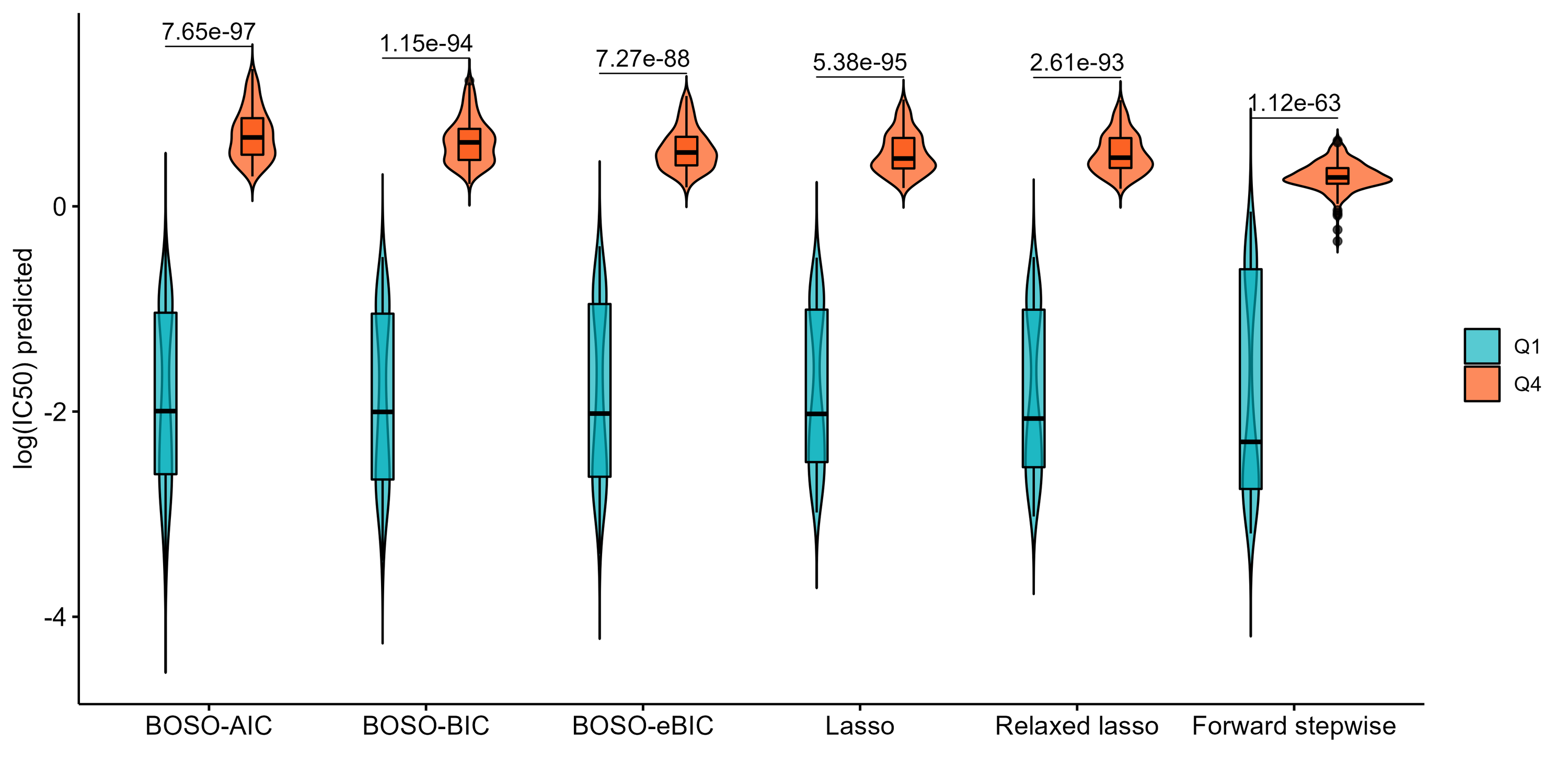

Supplement: S43 Fig — IC50 for each cell line were predicted using the mean value across 100 runs considered in Fig 7. Q1 involves cell lines with a predicted IC50 below the first quartile (sensitive cell lines), whereas Q4 cell lines with a predicted IC50 above the third quartile (resistant cell lines). In order to avoid overfitting, we considered 708 cell lines in CCLE that were not included in the GDSC database. (TIF) [file pcbi.1010180.s056.tif]

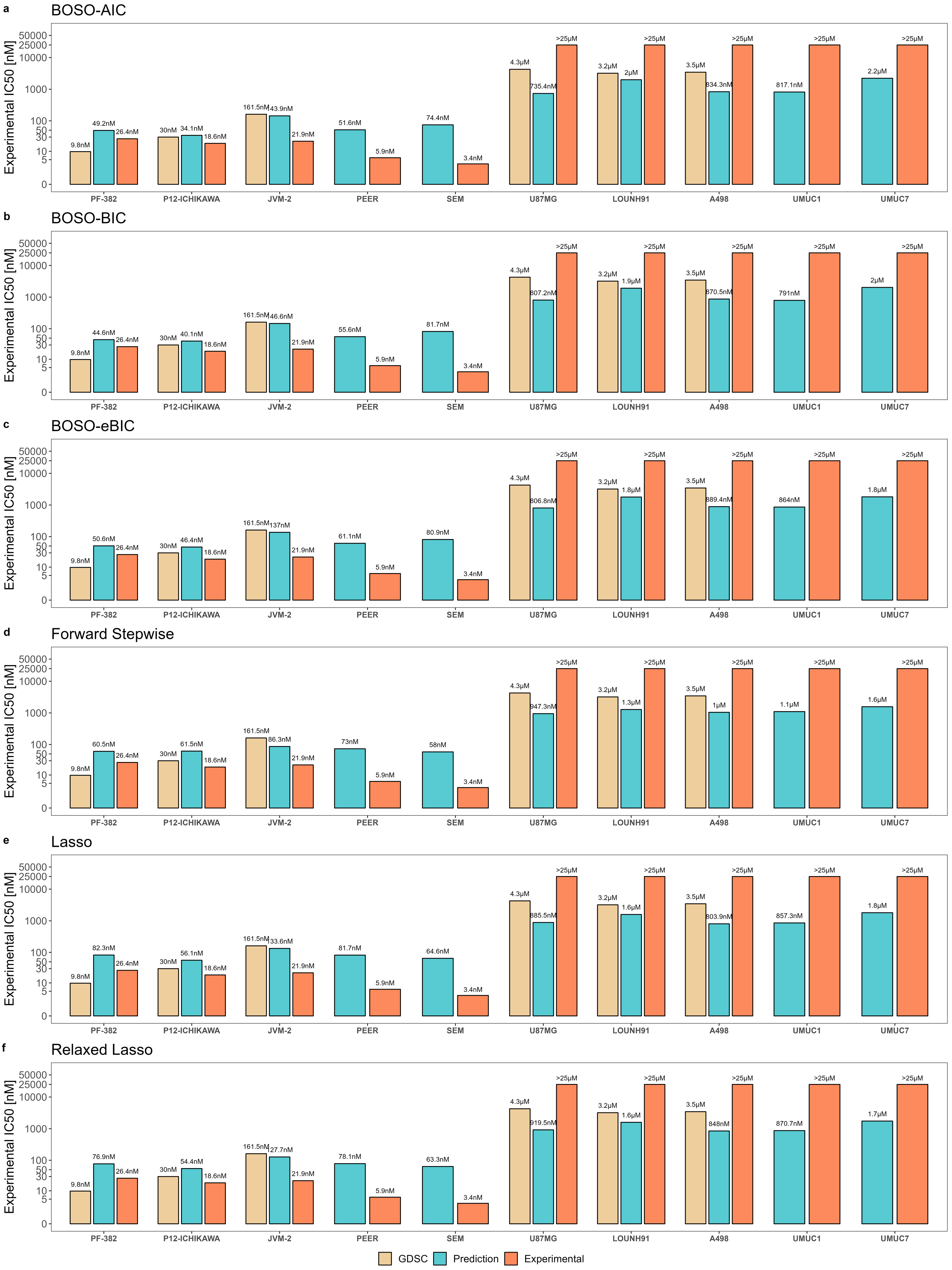

Supplement: S44 Fig — a) BOSO—AIC; b) BOSO—BIC; c) BOSO—eBIC; d) Forward Stepwise; e) Lasso; f) Relaxed Lasso. Predicted values are the mean values obtained with 100 random seeds. (TIF) [file pcbi.1010180.s057.tif]

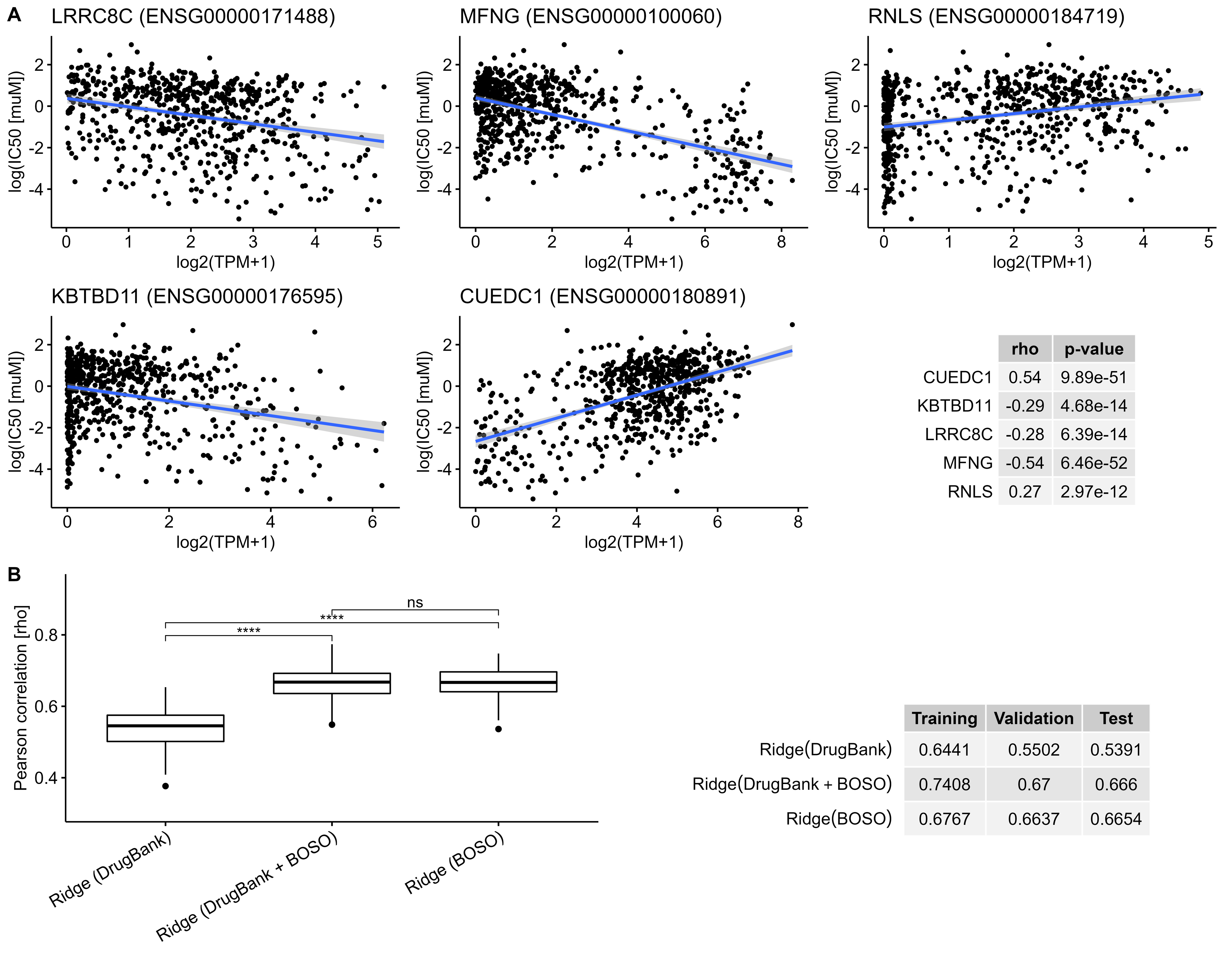

Supplement: S45 Fig — a) For each of the 5 best-ranked genes obtained from BOSO (LRRC8C, MFNG, RNLS, KBTBD11, CUEDC1), dot plot showing its corresponding CCLE expression level (x-axis) and MTX IC50 values (y-axis) for cell lines available in the GDSC database. The table shows the Pearson correlation rho value and its associated p-value for each these 5 genes. b) Ridge regression model of MTX IC50 value using as predictors i) genes annotated to MTX in DrugBank (see S12 Table), ii) 5 best-ranked genes obtained from BOSO and iii) the union of both subsets of genes. The table show the correlation between predicted and actual MTX IC50 values for training, validation and test set. (TIF) [file pcbi.1010180.s058.tif]
