# Supplementary material for: BOSO: A novel feature selection algorithm for linear regression with high-dimensional data
Source: PLoS Comput Biol. 2022 May 31;18(5):e1010180. doi: 10.1371/journal.pcbi.1010180 (PMC9187084; doi:10.1371/journal.pcbi.1010180)
Supplement: Attachment — Submitted filename: Response_Letter.docx [file pcbi.1010180.s059.docx]

*Reviewer #1:*

In this manuscript, Valcarcel et al. present a method called Bilevel Optimization Selector Operater (BOSO) to extract meaningful features in linear regression models. With an increase in high-dimensional data in Biology, it has become increasingly challenging to distill meaningful information to the bare minimum, that is sufficient, both for descriptive and predictive models. The identification of the most important variables is done via feature selection, one of the most commonly used tools in machine learning. Valcarcel et al. tackle this issue for linear regression models, and present BOSO as an algorithm that outperforms the other competing approaches in reducing dimensions, and selecting important features. The algorithm utilizes bilevel optimization model, where the split training and validation datasets are used for selection of optimal parameter set (minimize the loss function), and for selection of features with lowest validation error respectively. They perform multiple iterations of feature set, K, and select the best set that results in the optimum model selection criteria. They show that BOSO consistently compares to and/or outperforms the other algorithms in many aspects. They proceed to apply BOSO in predicting drug sensitivity in cancer datasets using RNASeq data. Overall, the prediction from BOSO is improved compared to other methods, while consistently selecting less number of features from the original dataset. Taken together, BOSO appears to generate accurate, and parsimonious models compared to other models for linear regression.

With an increase in multimodal measurements in biology, feature selection has become a critical step in understanding the complex biology, and using information from high-dimensional datasets in palatable way. Overall, I find the manuscript well-written, and easy to follow. However, several issues need to be addressed. My major concern is that I find that the conclusions regarding the superiority of BOSO compared to other methods misleading. BOSO, at most, is comparable to other methods, depending on the parameters used, and only in some instances superior, but very similar to other methods. Some major comments are listed below:

**Response:** We thank the reviewer for the feedback and kind words.

1. Authors stress that the use of validation datasets is novel. Can they show how not having a separate validation dataset undermines the predictive value of feature selection?

**Response:** Without the use of a validation dataset, it is not possible to unbiasedly tune the different parameters that are part of any machine learning model. This is necessary to fix, for example, the lambda parameter in Lasso regression. In our case, we used the validation set for feature selection, leading to a complex bilevel optimization problem, as detailed in the Methods section, Eqs. (1)-(4). The main novelty of BOSO comes from the reformulation of said bilevel optimization problem into a specific mixed-integer quadratic programming (BOSO MIQP), which can be globally solved with standard optimizers, such as IBM ILOG CPLEX. Thus, BOSO overcomes a difficult combinatorial problem and avoids heuristic strategies that would generate a large number of models with the training set, including different combinations of features, and then select the best one using the validation set.

Following the comment of reviewer, the role of validation data has been more clearly defined in the Results sections (see legend of Fig 1).

2. Authors argue BOSO reduces the computation time. Can they show the computation time differences for different methods used, and how BOSO compares to other methods?

**Response:** In the previous version of the manuscript, we included the following paragraph:

“With respect to computational effort, even using the random block strategy mentioned above, BOSO requires more time than Forward Stepwise, Lasso and Relaxed Lasso. However, BOSO is more efficient than Best Subset and can be run in standard computers, *e.g.* each run in the High-10 setting took us on average 104.6 seconds on a 64 bit Intel(R) Xeon(R) CPU E5-2630 v4 @ 2.20GHz running Linux, setting a maximum of 4 cores and 4 GB of RAM. Further details can be found in S1 Table.”

Thus, the differences in computation time were previously detailed in Supplementary Table 1, which can be observed below:

| Setting | **BOSO** | **Best Subset** | **Forward Stepwise** | **Lasso** | **Relaxed**  **Lasso** |
| --- | --- | --- | --- | --- | --- |
| **Low** | 6.041 | 0.692 | 0.014 | 0.003 | 0.003 |
| **Medium** | 73.029 | 17140.228 | 1.760 | 0.011 | 0.009 |
| **High-5** | 65.313 | 5444.814 | 0.334 | 0.013 | 0.006 |
| **High-10** | 104.607 | 10050.708 | 1.452 | 0.026 | 0.014 |

**S1 Table. Computation time in the benchmark with synthetic data**

For each setting (Low, Medium, High-5 and High-10), computation time (in seconds) was averaged across all cases considered (1200 cases: 4 beta-types, 3 autocorrelation levels, 10 SNR values and 10 random repetitions).

The misunderstanding may come from the following paragraph included in the subsection ‘The BOSO algorithm’ (Results section):

“The core MIQP of BOSO addresses a hard-combinatorial optimization problem, whose complexity exponentially grows as p increases. Current MIQP solvers have been widely developed in the last decade [24]; however, in the case of BOSO, for large problems, they could take long computation times to guarantee optimality. This is also the case for the MIQP approach presented in Bertsimas et al, 2016 [17], referred to here as Best Subset. Here, we alleviated this issue by iteratively applying BOSO to random blocks of features of length L until convergence (see Methods section and S1 Fig.). With this strategy, we substantially reduced the computation time and managed to apply BOSO to complex problems.”

With the sentence in the above paragraph “With this strategy, we substantially reduced the computation time and managed to apply BOSO to complex problems“, we were referring to our own approach. For the sake of clarity, we updated this sentence as follows: “With this strategy, we substantially reduced the computation time of our approach and managed to apply BOSO to complex problems “.

In summary, our method is slower than Lasso, Relaxed Lasso and Forward Stepwise. However, we substantially outperform Best Subset algorithm, which belongs to the same family of feature selection methods based on mathematical programming (MIQP). As reflected in the Results section, this relevant reduction makes BOSO a computationally feasible approach in large-sized problems encountered in biomedical research.

*3. For comparison to other methods in Figs 2-6, for many metrics used, BOSO, at most, is comparable to, and not superior than other methods. Authors need to tone down their conclusion to agree with the results.*

**Response:** Among the figures mentioned by the reviewer, Fig 2, which represents F1-score for different scenarios, is the one that best summarizes the performance of the different methods analyzed. F1-score is a global measure of accuracy that integrates false positives, true positives and false negatives via precision and recall. In Figure 2, the superiority of BOSO in high-dimensional datasets (High-5 and High-10) in our opinion is clear. However, following the reviewer’s recommendation, we have toned down the conclusion at the end of page 11:

“According to these results, BOSO is overall more accurate than Best Subset, Forward Stepwise and Lasso and competes with Relaxed Lasso, finding comparable accuracy in low-to-medium-dimensional problems and superior results in high-dimensional scenarios.“

In this direction, we also updated a sentence in the abstract to clarify that we are more accurate in selecting features in high-dimensional datasets.

On the other hand, we included Figs 3-5 in order to assess how different methods behaves from different points of view: number of non-zeros, false positives and false negatives. Based on them, we summarized our conclusions at the end of subsection “Benchmarking of feature selection approaches” (Results section):

“In summary, for feature selection: 1) BOSO shows higher sensitivity than Best Subset and Forward Stepwise; 2) BOSO presents higher specificity than Lasso and Relaxed Lasso; 3) BOSO is a computationally feasible approach in large-sized problems encountered in biomedical research.“

We think that this is fully justified because BOSO has less false negatives (more true positives) than Best Subset and Forward Stepwise (Fig 5) and BOSO has less false positives (more true negatives) than Lasso and Relaxed Lasso (Fig 4). This idea is also mentioned in the Discussion section because it summarized the behavior found with BOSO in the different cases analyzed.

*4. For the use of BOSO in RNASeq data, the discrepancy in model-calculated IC50 and experimental data is huge. It seems to me that BOSO consistently deflates the difference between sensitive, and insensitive cell-lines (Fig. 7d). Compared to experimental data, the prediction suggests that sensitivity is much lower for insensitive cells, while higher for sensitive cells.*

**Response:** This seems to be the case with the results presented in Fig 7d (now Fig 7c). However, we think this is due to the screening concentration range experimentally tested for Methotrexate in the GDSC database. It can be observed in Figure R1 that the maximum concentration tested is 0.2 µm (200 nm). IC50 values beyond this threshold (in red in Figure R1) were not experimentally tested and, thus, they are less reliable. As recognized by the authors in their website: “For completeness we have reported these IC-values but they should be interpreted carefully because the confidence in the reported value is reduced.”

**Figure R1: Summary of Methotrexate IC50 values in the GDSC database**

*5. Based on the results, it seems that some methods are better at predicting sensitivity for insensitive cells, while others are better in predicting sensitivity for sensitive cells. Can authors comment on why that is, and how that information could be used to optimize a better predictive model?*

**Response:** We would like to thank the reviewer for this question. Although it might appear that the methods considered obtain different solutions, it can be observed in Figure R2 that they predict similar IC50 value ranges for the top 25% most sensitive (Q1) and 25% most resistant (Q4) cell lines. The only exception is Forward Stepwise, which obtains higher MTX IC50 values than the rest methods in Q1 but also in Q4. Note here that Forward Stepwise is the least accurate method tested, according to the results presented in Fig 7a.

**Figure R2: Boxplot of predicted log(IC50[µM]) for the top 25% most sensitive and resistant cell lines with the different methods included in the main text**. IC50 for each cell line were predicted using the mean value across 100 runs considered in Fig 7. Q1 involves cell lines with a predicted IC50 below the first quartile (sensitive cell lines), whereas Q4 cell lines with a predicted IC50 above the third quartile (resistant cell lines). In order to avoid overfitting, we considered 708 cell lines in CCLE that were not included in the GDSC database.

*6. Authors state that there is "a significant agreement between computational predictions, and in-vitro experiments." The way the data is presented, it does not seem to have a trend that is consistent for both sensitive and insensitive cell-lines. Can authors elaborate on how they conclude that this is a significant agreement? Some sort of statistical tests might be necessary here.*

**Response:** Following the reviewer’s advice, we have conducted a comparison of MTX IC50 values between the top 25% most sensitive and resistant cell lines using the predictions presented in Figure R2. It can be observed in the figure below (now S43 Fig) that BOSO predicted statistically significant differences between these 2 groups of cell lines (t-test p-value = 4.29e-95 for BOSO-BIC). This analysis has been included in the main text (see last paragraph of page 14).

On the other hand, we compared the predicted IC50 values for the 10 cell lines where we conducted our own *in-vitro* experiments: 5 MTX-resistant cell lines and 5 MTX-sensitive. We have rephrased the sentence indicated by the reviewer as follows: *“Our approach could distinguish between these in-vitro validated MTX-resistant and MTX-sensitive cell lines (Student’s t-test p-value: 4.21e-5), indicating that the linear regression model derived by BOSO can be applied to complete the data provided by the GDSC database.”.* Note here that to in order to carry out this study unbiasedly, we did not include in the process of model construction the 6 cell lines presented in Fig 7c that are available in GDSC. For this reason, the accuracy results and Fig 7a-b were slightly modified. In addition, Fig 7c-d were integrated in Fig 7c.

**S43 Fig:** **Comparison of predicted log(IC50[µM]) for the top 25% most sensitive and resistant cell lines with the different methods included in the main text.** IC50 for each cell line were predicted using the mean value across 100 runs considered in Fig 7. Q1 involves cell lines with a predicted IC50 below the first quartile (sensitive cell lines), whereas Q4 cell lines with a predicted IC50 above the third quartile (resistant cell lines). In order to avoid overfitting, we considered 708 cell lines in CCLE that were not included in the GDSC database.

*7. While I agree that feature selection is necessary, reducing the number of features to a very small number might make the model less predictive. To that end, how minimum should the features be? By reducing the features, are we getting rid of valuable information that would otherwise be important for biological processes?*

**Response:** The reviewer has brought up a relevant question in the field. In our opinion, this obviously depends on the dataset and the problem analyzed. However, feature selection is a crucial strategy in high-dimensional biomedical questions with limited sample size in order to avoid overfitting in regression models. This has been now emphasized in the Introduction and Discussion section.

*Some minor comments that I have are outlined below:*

*1. Introduction needs a little more clarity. For instance, "Dimensionality reduction and feature selection selection are the most commonly used strategies to address this issue". What issue? And the transition to linear regression models in the next need to be smoother, as it is, it comes out of nowhere.*

**Response:** We have extended/rephrased the Introduction section to clarify the issue indicated by the reviewer. See the first 2 paragraphs of the Introduction section.

*2. For gene expression data, can authors elaborate more on how they filtered out the genes with low mean and variance expression? IN methods, the description is not detailed.*

**Response:** This was previously explained in the Methods section, page 18:

*“****Drug sensitivity in cancer***

*For the drug sensitivity analysis, RNA-seq data for different CCLE cancer cell lines was downloaded* from *the DepMap (Dependency Map) portal (*[*www.depmap.org*](http://www.depmap.org)*)[33]. Gene expression levels are provided in log2(TPM+1). We kept for further analysis those genes with: 1) mean expression value across the cell lines greater than 1 TPM; 2) variance across the cell lines greater than one unit. IC50 values were also taken from the DepMap portal.”*

*Reviewer #2:*

*The proposed BOSO (Bilevel Optimization Selector Operator) is a novel feature selection approach for linear regression. The method is novel and the data analysis using this method shows its advantage compared with other methods, including relaxed LASSO.*

*The paper is well written and organized, the method is sound, the data analysis is solid. In the Method description, I would like the authors to discuss how to find the optimal values for the parameters M, delta, etc, since these parameters will influence the feature selection results. In all, this is a good work.*

**Response:** We appreciate the positive comments of the reviewer.

First, we avoid selecting the value of M using indicators constraints available in IBM ILOG CPLEX. Indicator constraints internally deal with the selection of M value in order to overcome numerical issues [1]. This was previously indicated in the text. The reviewer can find a description of indicator constraints here:

Second, the K parameter, the optimal number of variables, is selected using eBIC or any other information criterion. We iteratively solve the problem until obtaining a minimum value in the implemented information criterion (AIC, BIC or eBIC), as it is explained in Figure 1.

Finally, the value of delta parameter is extracted as part of the solution of BOSO MILP. Delta can take a finite number of values that ranges between the minimum and maximum value of the lambda parameter involved in Lasso and Ridge regression, respectively, implemented in the *glmnet* R package [2]. The rest of δ values are equally spaced between the minimum and maximum value in a logarithmic scale. This has been included in the text (see Page 24).

[1]. https://www.ibm.com/docs/en/icos/20.1.0?topic=optimization-what-is-indicator-constraint.
[2]. Friedman, J., Hastie, T., & Tibshirani, R. (2010). Regularization paths for generalized linear models via coordinate descent. *Journal of statistical software*, 33(1), 1.

*Reviewer #3:*

*Valcarcel et al. present BOSO (Bilevel Optimization Selector Operator), a novel method to conduct feature selection in linear regression models. The authors have demonstrated the performance of their method with extensive testing on synthetic data as well as modeling the methotrexate sensitivity of cancer cell lines. The authors have also performed experimental validation of their methotrexate sensitivity predictions for 4 cell lines not included in their training data set. The value of this new approach is two-fold: improved performance (in some but not all synthetic data sets) and increased parsimony (fewer features). The manuscript is mostly focused on analysis of synthetic data, and as such the authors have shown methodological novelty. However, it would be nice to see the authors demonstrate that BOSO can provide new biological insights. Along those lines, there are several issues which warrant further explanation from the authors:*

*- Page 12: The authors claim that “BOSO obtained the best solution in test data with BIC: mean correlation of 0.628; 2) the models derived from Lasso and Relaxed Lasso have similar mean correlation in test data: 0.636 and 0.631, respectively;”. How is the BOSO solution “best” if the mean correlation of Lasso and Relaxed Lasso is larger than for BOSO?*

**Response:** We thank the reviewer for his/her thorough revision of our work. In this point we did not explained well enough the observation that the best performance of BOSO was obtained with BIC over eBIC or AIC. Indeed, Lasso and Relaxed Lasso were slightly more accurate than BOSO in this dataset. We have reformulated the sentence in the manuscript to clarify this point (see Page 13).

Note here that, in response to one of the questions of Reviewer 1, we deleted from the model construction process the 6 cell lines, available in the GDSC database, for which we had conducted *in-vitro* validation of Methotrexate IC50 values: PF-382, P12-ICHIKAWA, JVM-2, U87MG, A498, LOUNH91. For this reason, the accuracy results mentioned by the reviewer have been slightly modified (see Page 13).

- Page 13: In the predictive model of MTX IC50 value for “603 cell lines not included in the GDSC database”, what data was included in the training of this BOSO model? Were all 646 cancer cell lines with MTX IC50 values (GDSC) and RNA-seq data (CCLE) used? Or were the data randomly grouped into training (40%), validation (40%) and test (20%) sets like in Figs. 7a and 7b?

**Response:** The 646 cell lines of CCLE with available MTX IC50 data in the GDSC database (now updated to 662 cell lines) were randomly grouped into training (40%), validation (40%) and test (20%). This process was repeated 100 times in order to build 100 different regression models with training and validation data and evaluate their predicting power with test data. In Figs 7a-b, we show the boxplot of the correlation between the actual and predicted MTX IC50 values in the test partition for the 100 predictive models with the different methods considered.

Using the 100 predictive models described above, we obtained 100 predictions for the remaining cell lines in CCLE without information in the GDSC database (before 603 cell lines, now updated to 708). The MTX IC50 value reported for each of these cell lines corresponds to the mean value across the predictions obtained from said 100 predictive models. Thus, yes, we used the same trained BOSO model as in Figs 7a-b. We have clarified this point in the last paragraph of page 14.

- Page 13: In the predictive model of MTX IC50 values for “603 cell lines not included in the GDSC database”, what features were selected by BOSO? The authors claim that BOSO will simplify the interpretation of predictive models by selecting fewer features (see Fig. 7b). Can the authors provide some interpretation of their MTX model? Can the selected features be placed in context of known literature (i.e., the known target of MTX is dihydrofolate reductase), or have the authors uncovered novel predictors of methotrexate sensitivity?

**Response:** We would like to thank the reviewer for this interesting question. Surprisingly, the most relevant features in BOSO, according to their recurrence in different runs (see Supplementary Data), are not typically annotated to MTX in drug databases. In particular, the top-5 genes are: LRRC8C, MFNG, RNLS, KBTBD11 and CUEDC1. The individual expression of each gene exhibits a high and significant correlation with MTX IC50, as now shown in S45a Fig. Importantly, a linear model with these 5 genes substantially outperforms a model including the 30 genes annotated to MTX in DrugBank (S45b Fig, S12 Table), which shows the relevance of the novel predictors identified.

**S45 Fig. Summary of 5 best-ranked features in BOSO and accuracy comparison with features extracted from DrugBank.** A) For each of the 5 best-ranked genes obtained from BOSO (LRRC8C, MFNG, RNLS, KBTBD11, CUEDC1), dot plot showing its corresponding CCLE expression level (x-axis) and MTX IC50 values (y-axis) for cell lines available in the GDSC database. The table shows the Pearson correlation *rho* value and its associated p-value for each these 5 genes. B) Ridge regression model of MTX IC50 value using as predictors i) genes annotated to MTX in DrugBank (see S12 Table), ii) 5 best-ranked genes obtained from BOSO and iii) the union of both subsets of genes. The table show the correlation between predicted and actual MTX IC50 values for training, validation and test set.

The importance of these 5 genes in MTX resistance deserves further study and experimentation. However, existing literature provides promising insights about their potential mechanism of action. LRRC8C is a component of the volume-regulated anion channel (VRAC) that has been recently linked to multidrug resistance in cancer in compounds such as cisplatin [30]. MFNG is a manic fringe protein that regulates Notch signaling [31], a pathway previously associated with MTX resistance [32]. KBTBD11 is a tumor suppressor gene that has been identified as differentially expressed in MTX-resistant colon cancer cell lines [33]. CUEDC1 is correlated with estrogen receptor alpha (ERα) [34], which has been found to confer MTX resistance in osteosarcoma cells [35].

We have included this analysis in the Results section (see page 16) and mentioned their relevance in the Discussion section (see page 17).

- In “Supplementary Figure 42: Prediction of IC50 values for 50 drugs present in the GDSC database”, what drugs are being tested? I could not find this information, though perhaps it is buried in the Supplemental Data.

**Response:** The comment of the reviewer is pertinent. We have now included an additional supplementary table (S8 Table), which details drugs and number of cell lines for which the IC50 is available in the GDSC database.

**S8 Table. Details of 50 drugs in the GDSC database used to compare different feature selection algorithms.** Columns indicate the Drug Name and the number of cell lines for which the IC50 is available in the GDSC database.

| **Drug Name** | **Number of cell lines** |
| --- | --- |
| GEMCITABINE | 1179 |
| APO866, FK866 | 895 |
| VINCRISTINE | 537 |
| THZ-2-102-1 | 637 |
| OSI-027 | 853 |
| TRAMETINIB | 1197 |
| DASATINIB | 801 |
| DOCETAXEL | 1741 |
| AZD5582 | 533 |
| MITOXANTRONE | 539 |
| PI-103 | 642 |
| TOZASERTIB | 282 |
| ACETALAX, OXYPHENISATIN ACETATE | 539 |
| BLEOMYCIN | 614 |
| PACLITAXEL | 804 |
| THZ-2-49 | 642 |
| GSK1070916 | 620 |
| SNX-2112 | 645 |
| CYTARABINE | 1221 |
| WH-4-023 | 238 |
| CAMPTOTHECIN | 594 |
| TENIPOSIDE | 539 |
| NSC-319726 | 622 |
| YM-155 | 1182 |
| TALAZOPARIB | 1196 |
| IRINOTECAN | 588 |
| FLUDARABINE | 539 |
| FILANESIB | 641 |
| VINORELBINE | 1190 |
| ALISERTIB | 1179 |
| DABRAFENIB | 1175 |
| ABT-737 | 558 |
| WYE-125132 | 646 |
| TOPOTECAN | 539 |
| PHA-793887 | 650 |
| BLEOMYCIN (50 UM) | 657 |
| BI-2536 | 280 |
| THAPSIGARGIN | 613 |
| OTX015 | 539 |
| APR-246 | 542 |
| PD-0325901 | 1247 |
| TORIN-2 | 621 |
| PEVONEDISTAT | 1062 |
| DACTINOMYCIN | 1089 |
| CX-5461 | 646 |
| VINBLASTINE | 1225 |
| 5-FLUOROURACIL | 1240 |
| PALBOCICLIB | 1202 |
| ETOPOSIDE | 630 |
| NAVITOCLAX | 1222 |

- In “Supplementary Figure 42: Prediction of IC50 values for 50 drugs present in the GDSC database”, the mean Pearson correlation coefficient is roughly equivalent for the five methods tested (0.44 – 0.49). I’m worried that averaging the correlation coefficients across all 50 drugs could be confounding the results. If the authors consider each of the 50 drugs individually, how often does BOSO outperform the other numbers? And if the results are mixed (sometimes BOSO is better, sometimes worse), is there any rhyme or reason to when BOSO works best?

**Response:** The reviewer makes here an interesting question. As suggested by the reviewer, we calculated how many times BOSO outperforms the rest of the methods for the different drugs considered. BOSO obtained the highest correlation in the test partition in 18% cases, Lasso in 68% and Relaxed Lasso in 14% of the cases. In no case Forward Stepwise turned out to be the winner.

Having these results into account, we compared the difference of the Pearson correlation for each drug in the test partition between BOSO, Lasso and Relaxed Lasso. The median absolute difference of BOSO versus Lasso and Relaxed Lasso is of 1.5%, representing a median ratio of the correlation across drugs of 1.02, which, in our opinion, is not very relevant. In fact, these differences were only statistically significant in 8 out 50 drugs (with the above effect size), following a two-sided Student’s t-test. In contrast, the median ratio of variables used by BOSO in comparison to Lasso and Relaxed Lasso is 0.15 and 0.23, respectively, which, in our opinion, is very relevant.

In conclusion, similarly to the MTX case discussed in Results section, BOSO provides a similar solution to the one provided by Lasso in terms of accuracy, but using a significantly smaller fraction of the variables. For these reasons, we think that BOSO is a better choice for feature selection by substantially reducing the number of false positives, as it is also shown in the controlled synthetic dataset.

Minor points:

- there is a random “S” at the end of the author summary

- page 7, “presentedin”

**Response:** These typos have been corrected in the main text. Thanks.
